# PhI(OAc)_2_-mediated intramolecular oxidative C–N coupling and detosylative aromatization: an access to indolo[2,3-*b*]quinolines†

**DOI:** 10.1039/d1ra01894a

**Published:** 2021-05-10

**Authors:** Quan-Bing Wang, Shi Tang, Ying-Jie Wang, Yue Yuan, Tieqiao Chen, Ai-Qun Jia

**Affiliations:** School of Life and Pharmaceutical Sciences, Key Laboratory of Tropical Biological Resources of Ministry of Education, Hainan University Haikou 570228 China tangshi705@163.com ajia@hainanu.edu.cn

## Abstract

A PIDA mediated intramolecular oxidative C–N coupling and subsequent detosylative aromatization to afford indolo[2,3-*b*]quinoline derivatives has been developed. This tandem reaction provided an efficient method for the synthesis of valuable indolo[2,3-*b*]quinoline derivatives.

## Abstract 2

Under mild conditions, PIDA mediated oxidant C–N coupling to afford indolo[2,3-*b*]quinoline derivatives has been developed with a decent substrate scope.

## Graphical abstract

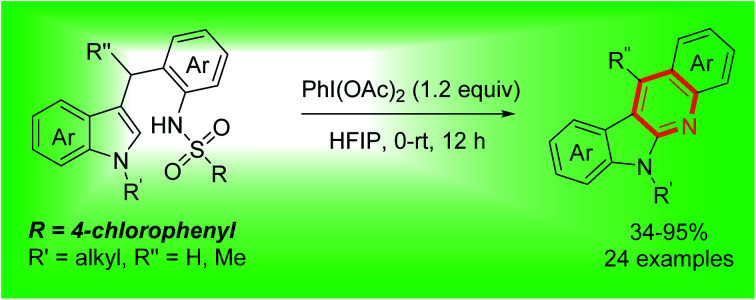

Indolo[2,3-*b*]quinolones are a kind of important poly-fused heterocycle, commonly occurring in many natural products and synthetic drugs (Fig. 1).^1^ These compounds usually display diverse biological activities^2^ such as anticancer,^3^ antiplasmodial,^4^ molluscicidal,^5^ and antimalarial activity^6^*etc.* Due to their importance, their efficient synthesis attracts chemists' interest and many synthetic methods have been developed.^7^ Among these established methods, indolo[2,3-*b*]quinolones are usually prepared through construction of a pyridine cycle with indole derivatives as the starting materials catalysed by transition metals.^8^ The more attractive metal-free methods avoiding the metal contamination of products have also been reported.^9^ Initially, indole-3-aldehydes or *o*-amino benzaldehydes were used; these compounds were usually unstable and difficult to prepare. In 2012, Liang and co-authors reported an efficient synthesis from indoles and *o*-sulfamidoaryl ketones.^10^ This reaction is a two-step process involving iodine-promoted amination/intramolecular cyclization and subsequent detosylation in 12 M HCl. The direct intramolecular cyclization of indole derivatives through oxidative C–N coupling and detosylative aromatization was also reported. In 2016, Sekar and co-authors pioneering used Ts (4-benzenesulfonyl) as the activating group for amino group under heat (Scheme 1A).^11^ In this reaction, 1.2 equiv. sublimed and corrosive I_2_ was used as the oxidant and 2 equiv. Cs_2_CO_3_ as the base, only 5 examples were demonstrated. Very recently, using the special Ns (4-nitrobenzenesulfonyl) to activate the amino group, Ishihara and co-authors achieved the reaction at room temperature through iodine catalysis (Scheme 1B).^12^ This reaction required 3–5 equiv. hazardous TBHP as the oxidant and relatively long reaction time (12–48 h).

**Fig. 1:**
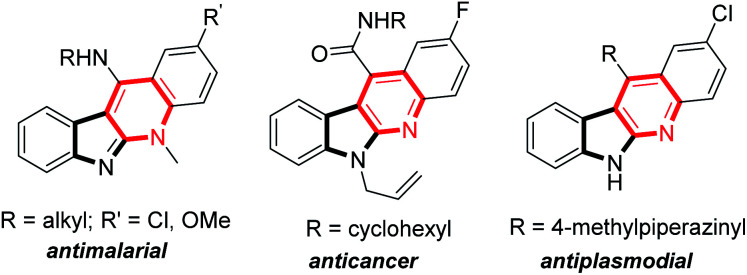
Selected examples of bioactive indolo[2,3-*b*]quinoline derivatives.

**Scheme 1:**
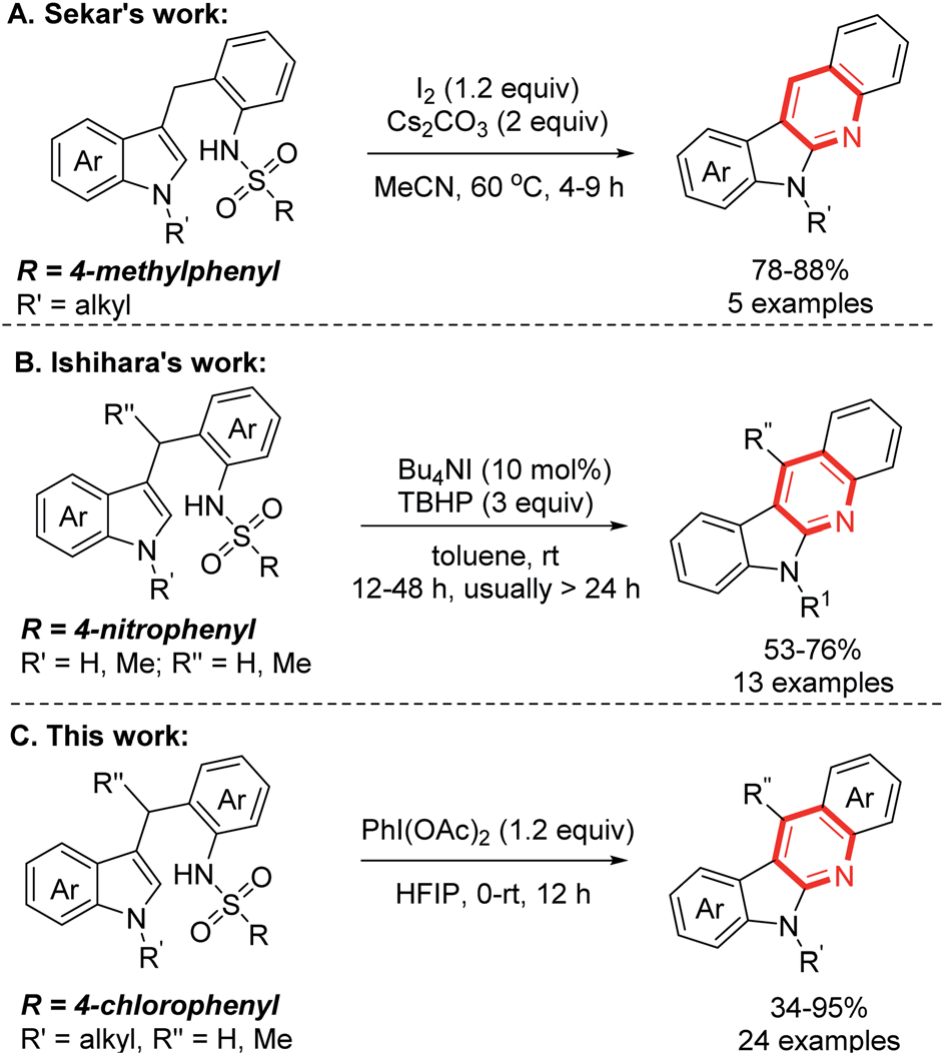
Metal-free intramolecular cyclization for preparing indolo[2,3-*b*]quinolones.

PhI(OAc)_2_ is a common oxidant used in annulation reactions due to its easy operation and environmental benignity.^13^ With our continuing interest in exploding synthesis of heterocyclic compounds,^14^ we envisioned that this reaction might also be achieved with the use of PhI(OAc)_2_ as the oxidant. Indeed, it worked well under the mild reaction conditions with the amino group being activated by 4-chlorobenzenesulfonyl group (Scheme 1C).

Stirring a mixture of 1a and 1.2 equiv. PhI(OAc)_2_ in DCM at room temperature for 12 h under an Ar atmosphere gave the product 2a in 10% yield (Table 1, entry 1). While using PhI(TFA)_2_ as the oxidant, only a trace amount of product was detected (entry 2). This reaction could proceed in other solvent, especially, 67% yield of 2a was obtained in HFIP (entries 3–7). Further increasing the loading of oxidant PhI(OAc)_2_ to 2 equiv. lead to decrease of the yield (entry 8, 15%). When the reaction was conducted at 0 °C, a low yield was given (entry 9, 49%). To our delight, by mixing the reactants at 0 °C and then stirring at room temperature, 2a was generated in a high yield (entry 10, 82%). The substrate 1b with 4-chlorobenzenesulfonyl group also worked well under the current reaction conditions, producing the cyclizing product 2a in 86% yield (entry 11). Using iodobenzene as a precursor of oxidant, screening of oxidant and solvent revealed that the desired product were not generated in a better yield (see the ESI† for details).

**Table 1:** Optimization of the reaction conditions^a^

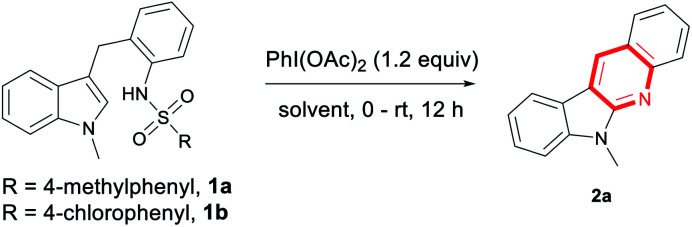			
Entry	Temp. (°C)	Solvent	Yield^b^ (%)
1	rt	DCM	10
2^c^	rt	DCM	Trace
3	rt	THF	28
4	rt	Dioxane	19
5	rt	PhCF_3_	16
6	rt	TFE	51
7	rt	HFIP	67
8^d^	rt	HFIP	15
9	0	HFIP	43
10	0–rt	HFIP	82
11^e^	0–rt	HFIP	86

a Reaction condition: 1a (0.2 mmol), PhI(OAc)_2_ (0.24 mmol), solvent (2 mL), rt, Ar, 12 h.

b Isolated yield.

c PhI(TFA)_2_ was used instead.

d 0.4 mmol PhI(OAc)_2_.

e 1b was used instead.

With the optimal reaction conditions in hand, we then investigated the substrate scope (Table 2). Various indole derivatives underwent the intramolecular oxidative C–N coupling/detosylative aromatization to produce the corresponding indolo[2,3-*b*]quinolones in moderate to high yields. Thus, in addition to 1a, the substrates with indole *N*-ethyl or benzyl group also gave the expected products in good yields (2b 67%, 2c 56%). It seems that both steric hindrance and electronic effect effected the reaction. For example, compared with C6-methylated indole (2g 61%), relatively low yields were given with steric C4 and C7-methylated ones (2d 52%, 2h 34%). Especially, a very high yield was obtained when the electron-rich C4-methoxylated indole was used (2e 93%). C5-chlorilated indole derivative also worked to produce 2f in 43% yield. Under the reaction conditions, substrates bearing functional groups at the aniline moiety also served well (2i–2o, 37–55%). As exemplified as 2p, the product bearing Me at C11 position was generated in 48% yield. Selected examples on the products with two groups were also synthesized by the strategy in good to high yields (2q–2x, 60–95%). Worth noting is that the halo groups like F, Cl and Br survived well in the current system. Fluoride is well-known in medicine chemistry. Those groups could also be transformed easily into other functional groups *via* cross coupling. Thus, these results described above well demonstrated the potential application of this new reaction in the synthetic chemistry.

**Table 2:** PhI(OAc)_2_-mediated intramolecular oxidative C–N coupling/detosylative aromatization forming indolo[2,3-*b*]quinolines^a^

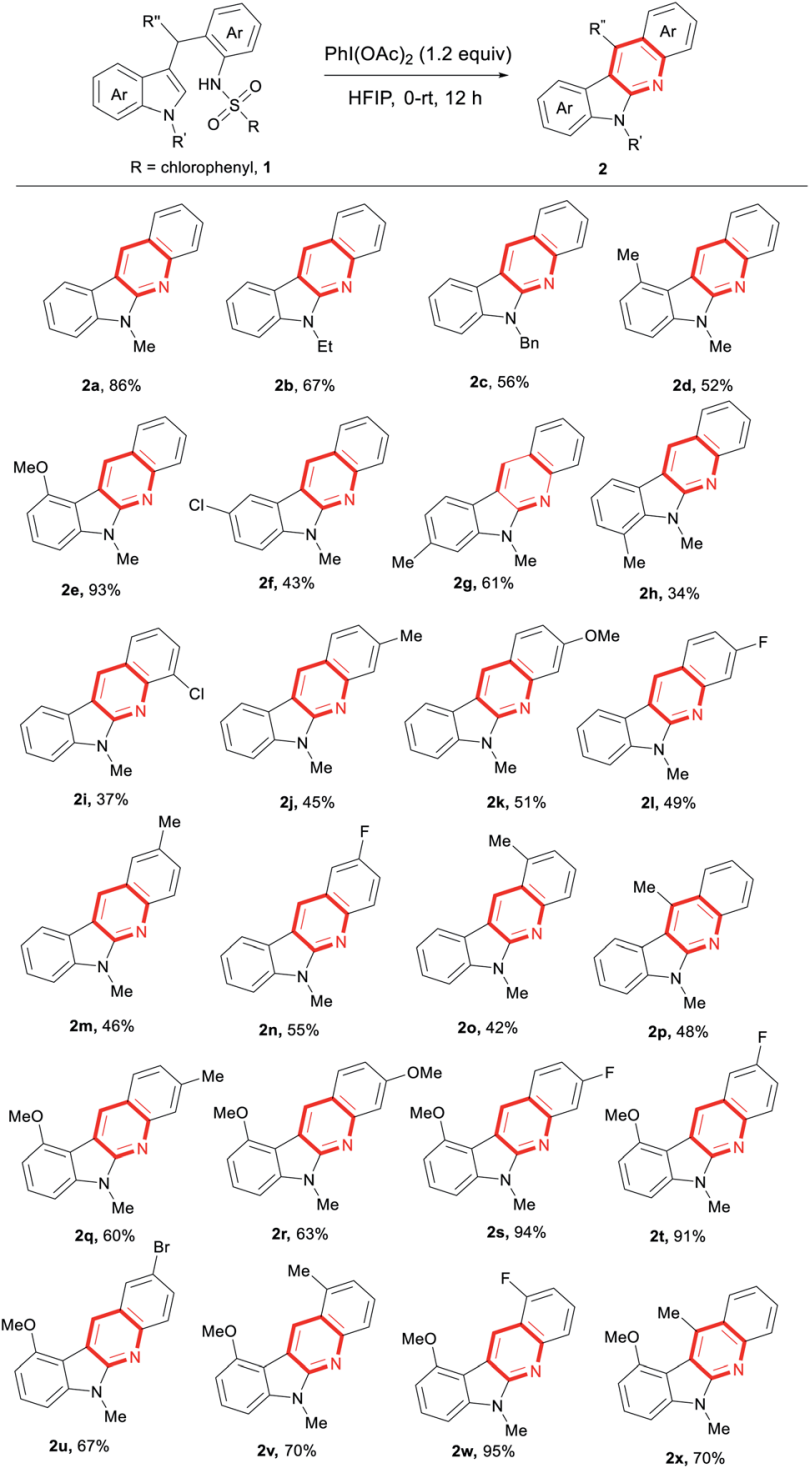

a Reaction condition: 1 (0.2 mmol), PhI(OAc)_2_ (0.24 mmol), HFIP (2 mL), at 0 °C, then temperature was increased to rt slowly and stirred for 12 h.

On the basis of previous literatures,^15^ a plausible mechanism for this intramolecular oxidative cyclization was proposed in Scheme 2. Initially, nucleophilic substitution of the amino nitrogen onto the iodine(iii) center in PIDA takes place to form intermediate A, followed by nucleophilic attack of the C3 or C2 position of indole, giving intermediate B or C and releasing AcO^−^ and PhI. The intermediate B was underwent rearrangement from C3 to C2 position to give intermediate C. Finally, two steps detosylative aromatization of intermediate C furnished the desired product 1a. Considering the steric effect and electronic effect, the process through nucleophilic attack of the C3 position of indole onto the nitrogen atom would be mainly pathway.

**Scheme 2:**
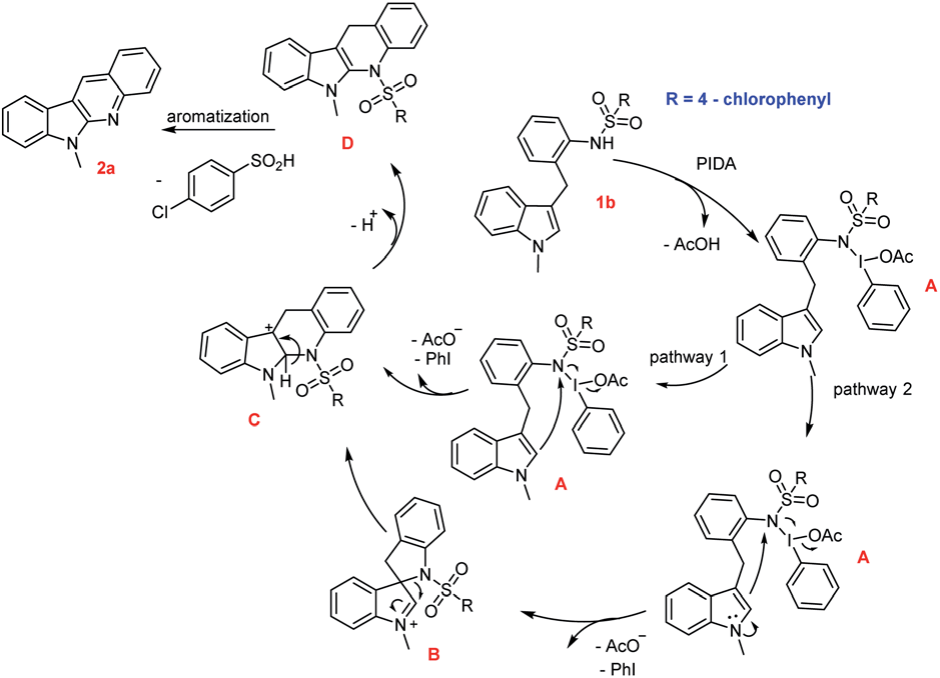
Proposed mechanism.

## Conclusions

In summary, we have developed a PhI(OAc)_2_-mediated oxidative cyclization forming indolo[2,3-*b*]quinolines. This reaction should take place through intramolecular oxidative C–N coupling and subsequent detosylative aromatization. By the strategy, a wide range of indolo[2,3-*b*]quinolines were readily produced in good to high yields. This reaction provides a facile and mild method to produce *N*-heterocyclic compounds.

## Experimental section

### General information

Reactions were monitored by using thin-layer chromatography (TLC) on commercial silica gel plates (GF 254). Visualization of the developed plates was performed under UV lights (GF 254 nm). Flash column chromatography was performed on silica gel (200–300 mesh). ^1^H and ^13^C NMR spectra were recorded on a 400 MHz spectrometer. Chemical shifts (*δ*) were reported in ppm referenced to the CDCl_3_ residual peak (*δ* 7.26) for ^1^H NMR. Chemical shifts of ^13^C NMR were reported relative to CDCl_3_ (*δ* 77.0). Infrared (IR) spectra was recorded on a Bruker TENSOR 27 FT-IR spectrometer. Melting points (mp) were taken on a MEL-TEMP® apparatus and are uncorrected. The following abbreviations were used to describe peak splitting patterns when appropriate: s = singlet, d = doublet, t = triplet, q = quartet, m = multiplet. Coupling constant, *J*, was reported in Hertz unit (Hz). High resolution mass spectra (HRMS) were obtained on an ESI-LC-MS/MS spectrometer.

### General procedure for synthesis of indolo[2,3-*b*]quinolines

To a mixture of 1 (0.2 mmol), PIDA (0.24 mmol), in Ar, at 0 °C, HFIP (2 mL) was added, then temperature was increased to rt slowly and stirred for 12 h. The solvent was removed under reduced pressure and was purified by silica gel flash column chromatography to afford the product 2.

### Characterization data for all products

#### 6-Methyl-6*H*-indolo[2,3-*b*]quinoline (2a)^16^

Pale yellow solid; 39.9 mg, 86% yield; ^1^H NMR (400 MHz, CDCl_3_) *δ* 8.72 (s, 1H), 8.17–8.13 (m, 2H), 8.01 (dd, *J* = 8.1, 1.2 Hz, 1H), 7.75–7.70 (m, 1H), 7.61–7.57 (m, 1H), 7.48–7.42 (m, 2H), 7.33–7.29 (m, 1H), 4.00 (s, 3H). ^13^C NMR (100 MHz, CDCl_3_) *δ* 152.96, 146.94, 142.99, 128.99, 128.67, 128.22, 127.61, 127.51, 124.24, 123.03, 121.56, 120.53, 120.08, 118.35, 108.85, 27.87.

#### 6-Ethyl-6*H*-indolo[2,3-*b*]quinoline (2b)^17^

Pale yellow solid; 32.9 mg, 67% yield; ^1^H NMR (400 MHz, CDCl_3_) *δ* 8.73 (s, 1H), 8.18–8.13 (m, 2H), 8.01 (dd, *J* = 8.1, 1.4 Hz, 1H), 7.74–7.70 (m, 1H), 7.60–7.56 (m, 1H), 7.48–7.44 (m, 2H), 7.32–7.28 (m, 1H), 4.61 (q, *J* = 7.2 Hz, 2H), 1.52 (t, *J* = 7.2 Hz, 3H). ^13^C NMR (100 MHz, CDCl_3_) *δ* 152.35, 146.99, 142.05, 128.89, 128.64, 128.13, 127.74, 127.41, 124.27, 122.97, 121.71, 120.74, 119.89, 118.44, 109.03, 36.28, 13.85.

#### 6-Benzyl-6*H*-indolo[2,3-*b*]quinoline (2c)^17^

Yellow solid; 34.5 mg, 56% yield; ^1^H NMR (400 MHz, CDCl_3_) *δ* 8.74 (s, 1H), 8.16–8.12 (m, 2H), 8.02 (d, *J* = 7.8 Hz, 1H), 7.75–7.67 (m, 1H), 7.47 (dd, *J* = 11.2, 4.0 Hz, 2H), 7.34–7.22 (m, 7H), 5.76 (s, 2H). ^13^C NMR (100 MHz, CDCl_3_) *δ* 152.86, 147.02, 142.23, 137.42, 128.96, 128.87, 128.62, 128.20, 127.87, 127.56, 127.49, 127.33, 124.54, 123.17, 121.60, 120.81, 120.28, 118.28, 109.84, 45.13.

#### 6,10-Dimethyl-6*H*-indolo[2,3-*b*]quinoline (2d)

Yellow solid; 25.6 mg, 52% yield; mp 135–137 °C. ^1^H NMR (400 MHz, CDCl_3_) *δ* 8.72 (s, 1H), 8.14 (d, *J* = 8.5 Hz, 1H), 8.01 (dd, *J* = 8.1, 1.4 Hz, 1H), 7.75–7.70 (m, 1H), 7.54–7.43 (m, 2H), 7.28 (s, 1H), 7.10 (d, *J* = 7.4 Hz, 1H), 3.98 (s, 3H), 2.93 (s, 3H). ^13^C NMR (100 MHz, CDCl_3_) *δ* 152.80, 146.28, 143.01, 134.87, 129.52, 128.92, 128.78, 127.97, 127.42, 124.31, 122.92, 121.79, 118.99, 118.96, 106.39, 27.92, 20.75. IR (KBr) 2923.03, 2852.53, 1602.82, 1570.67, 1479.48, 1393.82, 751.76 cm^−1^; HRMS (ESI) calcd for C_17_H_15_N_2_ [M + H]^+^: 247.1235, found: 247.1224.

#### 10-Methoxy-6-methyl-6*H*-indolo[2,3-*b*]quinoline (2e)

Yellow solid; 48.8 mg, 93% yield; mp 91–93 °C. ^1^H NMR (400 MHz, CDCl_3_) *δ* 8.88 (s, 1H), 8.14 (d, *J* = 8.5 Hz, 1H), 8.02 (dd, *J* = 8.1, 1.3 Hz, 1H), 7.73–7.69 (m, 1H), 7.53–7.48 (m, 1H), 7.47–7.43 (m, 1H), 7.03 (d, *J* = 8.0 Hz, 1H), 6.78 (d, *J* = 8.2 Hz, 1H), 4.13 (s, 3H), 3.97 (s, 3H). ^13^C NMR (100 MHz, CDCl_3_) *δ* 156.96, 152.48, 146.20, 144.27, 129.76, 129.13, 128.69, 128.57, 127.46, 124.73, 122.86, 117.52, 109.14, 101.82, 101.76, 55.72, 28.09. IR (KBr) 2925.15, 2851.85, 1603.61, 1500.60, 1473.37, 1392.59, 1345.15, 739.18 cm^−1^; HRMS (ESI) calcd for C_17_H_15_N_2_O [M + H]^+^: 263.1184, found: 263.1184.

#### 9-Chloro-6-methyl-6*H*-indolo[2,3-*b*]quinoline (2f)^17^

Pale yellow solid; 22.9 mg, 43% yield; ^1^H NMR (400 MHz, CDCl_3_) *δ* 8.67 (s, 1H), 8.13 (d, *J* = 8.6 Hz, 1H), 8.09 (d, *J* = 2.0 Hz, 1H), 8.00 (dd, *J* = 8.2, 1.2 Hz, 1H), 7.76–7.72 (m, 1H), 7.53 (dd, *J* = 8.6, 2.1 Hz, 1H), 7.49–7.45 (m, 1H), 7.33 (d, *J* = 8.6 Hz, 1H), 3.97 (s, 3H). ^13^C NMR (100 MHz, CDCl_3_) *δ* 152.92, 147.22, 141.26, 129.47, 128.79, 128.16, 128.13, 127.68, 125.54, 124.21, 123.34, 121.67, 121.43, 117.34, 109.81, 27.99.

#### 6,8-Dimethyl-6*H*-indolo[2,3-*b*]quinoline (2g)

Yellow solid; 30.0 mg, 61% yield; mp123–126 °C.^1^H NMR (400 MHz, CDCl_3_) *δ* 8.64 (s, 1H), 8.13 (d, *J* = 8.5 Hz, 1H), 8.04–7.97 (m, 2H), 7.72–7.68 (m, 1H), 7.47–7.43 (m, 1H), 7.22 (s, 1H), 7.13 (d, *J* = 7.8 Hz, 1H), 3.97 (s, 3H), 2.60 (s, 3H). ^13^C NMR (100 MHz, CDCl_3_) *δ* 153.18, 146.60, 143.38, 138.80, 128.74, 128.56, 127.53, 126.84, 124.29, 122.95, 121.36, 121.28, 118.49, 118.07, 109.31, 27.81, 22.57. IR (KBr) 2921.06, 2853.06, 1608.54, 1570.70, 1425.35, 1398.74, 750.86 cm^−1^; HRMS (ESI) calcd for C_17_H_15_N_2_ [M + H]^+^: 247.1235, found: 247.1233.

#### 6,7-Dimethyl-6*H*-indolo[2,3-*b*]quinoline (2h)^16^

Yellow solid; 16.7 mg, 34% yield; ^1^H NMR (400 MHz, CDCl_3_) *δ* 8.68 (s, 1H), 8.13 (d, *J* = 8.5 Hz, 1H), 8.04–7.96 (m, 1H), 7.73–7.69 (m, 1H), 7.47–7.43 (m, 1H), 7.29 (d, *J* = 7.3 Hz, 1H), 7.20–7.18 (m, 1H), 4.30 (s, 3H), 2.89 (s, 3H). ^13^C NMR (100 MHz, CDCl_3_) *δ* 153.45, 146.96, 141.46, 131.39, 128.86, 128.57, 127.63, 127.10, 124.33, 122.96, 121.13, 120.90, 120.17, 119.41, 118.37, 31.06, 20.02.

#### 4-Chloro-6-methyl-6*H*-indolo[2,3-*b*]quinoline (2i)

Yellow solid; 19.7 mg, 37% yield; mp 136–138 °C.^1^H NMR (400 MHz, CDCl_3_) *δ* 8.71 (s, 1H), 8.18–8.13 (m, 1H), 7.93 (dd, *J* = 8.2, 1.1 Hz, 1H), 7.85 (dd, *J* = 7.4, 1.4 Hz, 1H), 7.62 (m, 1H), 7.45 (d, *J* = 8.1 Hz, 1H), 7.39–7.31 (m, 2H), 4.05 (s, 3H). ^13^C NMR (100 MHz, CDCl_3_) *δ* 153.04, 143.26, 142.98, 131.49, 129.02, 128.67, 127.78, 127.75, 125.33, 122.62, 121.85, 120.40, 120.18, 118.91, 109.13, 28.00. IR (KBr) 2924.76, 1639.47, 1605.34, 1429.92, 1397.91, 1378.97, 745.54 cm^−1^; HRMS (ESI) calcd for C_16_H_12_ClN_2_ [M + H]^+^: 267.0689, found: 267.0689.

#### 3,6-Dimethyl-6*H*-indolo[2,3-*b*]quinoline (2j)

White solid; 22.1 mg, 45% yield; mp 117–119 °C. ^1^H NMR (400 MHz, CDCl_3_) *δ* 8.61 (s, 1H), 8.10 (dd, *J* = 7.7, 0.6 Hz, 1H), 7.93 (d, *J* = 0.7 Hz, 1H), 7.86 (d, *J* = 8.3 Hz, 1H), 7.60–7.52 (m, 1H), 7.38 (d, *J* = 8.1 Hz, 1), 7.33–7.26 (m, 2H), 3.95 (s, 3H), 2.61 (s, 3H). ^13^C NMR (100 MHz, CDCl_3_) *δ* 152.91, 147.07, 142.65, 139.18, 128.18, 127.75, 127.15, 126.65, 125.18, 122.16, 121.21, 120.56, 119.84, 117.45, 108.63, 27.67, 22.04. IR (KBr) 2924.87, 2854.20, 1623.55, 1605.65, 1473.34, 1390.25, 746.78 cm^−1^; HRMS (ESI) calcd for C_17_H_15_N_2_ [M + H]^+^: 247.1235, found: 247.1233.

#### 3-Methoxy-6-methyl-6*H*-indolo[2,3-*b*]quinoline (2k)

White solid; 26.7 mg, 51% yield; mp 157–159 °C. ^1^H NMR (400 MHz, CDCl_3_) *δ* 8.63 (s, 1H), 8.11 (d, *J* = 7.7 Hz, 1H), 7.87 (d, *J* = 8.9 Hz, 1H), 7.57–7.54 (m, 1H), 7.47 (s, 1H), 7.41 (d, *J* = 8.0 Hz, 1H), 7.32–7.28 (m, 1H), 7.12 (dd, *J* = 8.9, 2.3 Hz, 1H), 4.00 (s, 3H), 3.99 (s, 3H). ^13^C NMR (100 MHz, CDCl_3_) *δ* 160.82, 153.32, 148.76, 142.33, 129.69, 127.55, 127.54, 121.08, 120.86, 120.08, 119.37, 116.29, 116.22, 108.81, 106.03, 55.68, 27.88. IR (KBr) 2930.51, 2854.42, 1613.60, 1604.62, 1444.79, 1412.11, 1390.77, 743.54 cm^−1^; HRMS (ESI) calcd for C_17_H_15_N_2_O [M + H]^+^: 263.1184, found: 263.1186.

#### 3-Fluoro-6-methyl-6*H*-indolo[2,3-*b*]quinoline (2l)^16^

Yellow solid; 24.5 mg, 49% yield; ^1^H NMR (400 MHz, CDCl_3_) *δ* 8.67 (s, 1H), 8.15–8.10 (m, 1H), 7.96 (dd, *J* = 9.0, 6.3 Hz, 1H), 7.75 (dd, *J* = 10.9, 2.5 Hz, 1H), 7.59 (m, 1H), 7.42 (d, *J* = 8.1 Hz, 1H), 7.32 (m, 1H), 7.25–7.20 (m, 1H), 3.97 (s, 3H). ^13^C NMR (100 MHz, CDCl_3_) *δ* 164.45, 161.99, 153.48, 147.96 (d, *J* = 13.0 Hz), 142.76, 130.41 (d, *J* = 10.5 Hz), 128.24, 127.43, 121.44, 121.16, 120.49, 120.33, 113.48 (d, *J* = 26.0 Hz), 111.46 (d, *J* = 21.0 Hz), 108.98, 27.87.

#### 2,6-Dimethyl-6*H*-indolo[2,3-*b*]quinoline (2m)^17^

Yellow solid; 22.6 mg, 46% yield; ^1^H NMR (400 MHz, CDCl_3_) *δ* 8.62 (s, 1H), 8.14 (d, *J* = 7.6 Hz, 1H), 8.04 (d, *J* = 8.6 Hz, 1H), 7.76 (s, 1H), 7.60–7.54 (m, 2H), 7.41 (d, *J* = 8.1 Hz, 1H), 7.32–7.28 (m, 1H), 3.98 (s, 3H), 2.57 (s, 3H). ^13^C NMR (100 MHz, CDCl_3_) *δ* 152.54, 145.32, 142.84, 132.39, 131.18, 127.96, 127.83, 127.19, 126.75, 124.13, 121.38, 120.47, 119.78, 118.16, 108.65, 27.73, 21.44.

#### 2-Fluoro-6-methyl-6*H*-indolo[2,3-*b*]quinoline (2n)^17^

Yellow solid; 27.5 mg, 55% yield; ^1^H NMR (400 MHz, CDCl_3_) *δ* 8.62 (s, 1H), 8.15–8.08 (m, 2H), 7.63–7.57 (m, 2H), 7.52–7.46 (m, 1H), 7.41 (d, *J* = 8.1 Hz, 1H), 7.33–7.29 (m, 1H), 3.97 (s, 3H). ^13^C NMR (100 MHz, CDCl_3_) *δ* 159.70, 157.29, 152.67, 143.83, 143.21, 129.62 (d, *J* = 8.9 Hz), 128.68, 126.64 (d, *J* = 5.1 Hz), 124.34 (d, *J* = 9.5 Hz), 121.84, 120.15 (d, *J* = 12.7 Hz), 119.06 (d, *J* = 4.6 Hz), 118.83, 111.41 (d, *J* = 21.6 Hz), 108.96, 27.90.

#### 1,6-Dimethyl-6*H*-indolo[2,3-*b*]quinoline (2o)

Yellow solid; 20.7 mg, 42% yield; mp 101–103 °C. ^1^H NMR (400 MHz, CDCl_3_) *δ* 8.90 (d, *J* = 0.6 Hz, 1H), 8.19 (d, *J* = 8, 1H), 8.00 (d, *J* = 8.6 Hz, 1H), 7.64–7.56 (m, 2H), 7.43 (d, *J* = 8.1 Hz, 1H), 7.34–7.27 (m, 2H), 4.00 (s, 3H), 2.85 (s, 3H). ^13^C NMR (100 MHz, CDCl_3_) *δ* 152.61, 147.24, 143.04, 135.20, 128.70, 128.11, 126.12, 123.90, 123.86, 123.58, 121.46, 120.79, 120.01, 117.84, 108.86, 27.85, 19.64. IR (KBr) 2923.70, 2853.99, 1604.96, 1578.42, 1473.47, 1429.70, 1396.11, 737.75 cm^−1^; HRMS (ESI) calcd for C_17_H_15_N_2_ [M + H]^+^: 247.1235, found: 247.1238.

#### 6,11-Dimethyl-6*H*-indolo[2,3-*b*]quinoline (2p)^17^

Yellow solid; 23.6 mg, 48% yield; ^1^H NMR (400 MHz, CDCl_3_) *δ* 8.29 (d, *J* = 7.8 Hz, 1H), 8.25 (dd, *J* = 8.5, 0.9 Hz, 1H), 8.13 (dd, *J* = 8.5, 0.7 Hz, 1H), 7.74–7.70 (m, 1H), 7.60–7.56 (m, 1H), 7.51–7.47 (m, 1H), 7.43 (d, *J* = 8.1 Hz, 1H), 7.34–7.30 (m, 1H), 3.98 (s, 3H), 3.20 (s, 3H). ^13^C NMR (100 MHz, CDCl_3_) *δ* 152.40, 146.67, 142.84, 139.09, 128.72, 128.14, 127.45, 124.20, 124.18, 123.68, 122.71, 121.51, 119.98, 116.54, 108.63, 27.72, 15.25.

#### 10-Methoxy-3,6-dimethyl-6*H*-indolo[2,3-*b*]quinoline (2q)

Yellow solid; 33.1 mg, 60% yield; mp 109–112 °C. ^1^H NMR (400 MHz, CDCl_3_) *δ* 8.84 (s, 1H), 7.91 (s, 2H), 7.51–7.47 (m, 1H), 7.29 (dd, *J* = 8.2, 1.6 Hz, 1H), 7.03 (d, *J* = 8.0 Hz, 1H), 6.78 (d, *J* = 8.1 Hz, 1H), 4.13 (s, 3H), 3.97 (s, 3H), 2.60 (s, 3H). ^13^C NMR (100 MHz, CDCl_3_) *δ* 156.84, 152.62, 146.49, 144.12, 138.87, 129.62, 128.82, 128.34, 126.67, 125.17, 122.79, 116.81, 109.34, 101.83, 101.73, 55.73, 28.08, 22.13. HRMS (ESI) calcd for C_18_H_17_N_2_O [M + H]^+^: 277.1341, found: 277.1347.

#### 3,10-Dimethoxy-6-methyl-6*H*-indolo[2,3-*b*]quinoline (2r)

Yellow solid; 36.8 mg, 63% yield; mp 115–117 °C. ^1^H NMR (400 MHz, CDCl_3_) *δ* 8.80 (s, 1H), 7.87 (d, *J* = 8.9 Hz, 1H), 7.52–7.43 (m, 2H), 7.11 (dd, *J* = 8.9, 2.5 Hz, 1H), 7.03 (d, *J* = 8.0 Hz, 1H), 6.77 (d, *J* = 8.1 Hz, 1H), 4.12 (s, 3H), 4.00 (s, 3H), 3.96 (s, 3H). ^13^C NMR (100 MHz, CDCl_3_) *δ* 160.51, 156.60, 152.80, 148.00, 143.63, 129.78, 129.6, 128.41, 119.84, 115.93, 115.51, 109.48, 106.01, 101.84, 101.77, 55.69, 55.62, 28.08. IR (KBr) 2931.15, 2853.12, 1604.32, 1503.96, 1473.90, 1387.23, 1369.26, 766.52 cm^−1^; HRMS (ESI) calcd for C_18_H_17_N_2_O_2_ [M + H]^+^: 293.1290, found: 293.1221.

#### 3-Fluoro-10-methoxy-6-methyl-6*H*-indolo[2,3-*b*]quinoline (2s)

Yellow solid; 52.6 mg, 94% yield; mp 179–181 °C. ^1^H NMR (400 MHz, CDCl_3_) *δ* 8.84 (s, 1H), 7.97 (dd, *J* = 9.0, 6.4 Hz, 1H), 7.73 (dd, *J* = 11.0, 2.5 Hz, 1H), 7.53–7.49 (m, 1H), 7.26–7.19 (m, 1H), 7.04 (d, *J* = 8.0 Hz, 1H), 6.79 (d, *J* = 8.2 Hz, 1H), 4.13 (s, 3H), 3.96 (s, 3H). ^13^C NMR (101 MHz, CDCl_3_) *δ* 164.22, 161.76, 156.89, 153.04, 147.23 (d, *J* = 12.8 Hz), 144.06, 130.39 (d, *J* = 10.5 Hz), 129.68, 129.17, 121.66, 116.98, 113.25 (d, *J* = 100.8 Hz), 111.34 (d, *J* = 82.8 Hz), 109.15, 101.99 (d, *J* = 5.9 Hz), 55.76, 28.11. IR (KBr) 2924.96, 2853.47, 1608.08, 1507.65, 1472.03, 1392.11, 1356.36, 1344.94, 764.66 cm^−1^; HRMS (ESI) calcd for C_17_H_14_FN_2_O [M + H]^+^: 281.1090, found: 281.1091.

#### 2-Fluoro-10-methoxy-6-methyl-6*H*-indolo[2,3-*b*]quinoline (2t)

Yellow solid; 50.9 mg, 91% yield; mp 136–138 °C. ^1^H NMR (400 MHz, CDCl_3_) *δ* 8.75 (s, 1H), 8.09–8.05 (m, 1H), 7.59 (dd, *J* = 9.4, 2.9 Hz, 1H), 7.52–7.42 (m, 2H), 6.99 (d, *J* = 8.0 Hz,1H), 6.75 (d, *J* = 8.1 Hz, 1H), 4.11 (s, 3H), 3.92 (s, 3H). ^13^C NMR (100 MHz, CDCl_3_) *δ* 159.55, 157.12 (d, *J* = 4.0 Hz), 152.11, 144.39, 142.98, 129.49, 129.39 (d, *J* = 34.8 Hz), 128.71 (d, *J* = 5.2 Hz), 124.75 (d, *J* = 9.6 Hz), 118.44, 118.13 (d, *J* = 11.0 Hz), 111.42, 111.21, 108.71, 101.74 (d, *J* = 4.3 Hz), 55.70, 27.99. IR (KBr) 2926.65, 2840.94, 1606.67, 1502.76, 1419.20, 1411.92, 1391.61, 1341.22, 766.77 cm^−1^; HRMS (ESI) calcd for C_17_H_14_FN_2_O [M + H]^+^: 281.1090, found: 281.1091.

#### 2-Bromo-10-methoxy-6-methyl-6*H*-indolo[2,3-*b*]quinoline (2u)

Yellow solid; 37.5 mg, 67% yield; mp 211–213 °C. ^1^H NMR (400 MHz, CDCl_3_) *δ* 8.71 (s, 1H), 8.11 (d, *J* = 2.2 Hz, 1H), 7.96 (d, *J* = 9.0 Hz, 1H), 7.72 (dd, *J* = 9.0, 2.3 Hz, 1H), 7.51 (m, 1H), 7.01 (d, *J* = 8.0 Hz, 1H), 6.77 (d, *J* = 8.2 Hz, 1H), 4.12 (s, 3H), 3.92 (s, 3H). ^13^C NMR (100 MHz, CDCl_3_) *δ* 157.06, 152.43, 144.63, 144.34, 131.54, 130.38, 129.58, 129.13, 128.42, 125.79, 118.05, 115.82, 108.84, 101.90, 101.85, 55.72, 28.02. IR (KBr) 2933.33, 2831.62, 1605.38, 1508.34, 1453.98, 1406.79, 1388.84, 1341.55, 736.58 cm^−1^; HRMS (ESI) calcd for C_17_H_14_BrN_2_O [M + H]^+^: 341.0290, found: 341.0269.

#### 10-Methoxy-1,6-dimethyl-6*H*-indolo[2,3-*b*]quinoline (2v)

Yellow solid; 38.6 mg, 70% yield; mp 80–83 °C. ^1^H NMR (400 MHz, CDCl_3_) *δ* 9.02 (s, 1H), 8.00 (d, *J* = 8.5 Hz, 1H), 7.59 (dd, *J* = 8.5, 7.0 Hz, 1H), 7.50 (t, *J* = 8.1 Hz, 1H), 7.29 (d, *J* = 6.9 Hz, 1H), 7.03 (d, *J* = 8.0 Hz, 1H), 6.78 (d, *J* = 8.2 Hz, 1H), 4.14 (s, 3H), 3.97 (s, 3H), 2.85 (s, 3H). ^13^C NMR (100 MHz, CDCl_3_) *δ* 156.93, 152.06, 146.44, 144.29, 135.28, 128.97, 128.26, 126.15, 125.93, 123.99, 123.66, 117.08, 109.35, 101.82, 101.68, 55.76, 28.04, 19.70. IR (KBr) 2925.87, 2853.72, 1603.91, 1502.99, 1474.17, 1397.32, 1346.74, 738.96 cm^−1^; HRMS (ESI) calcd for C_18_H_17_N_2_O [M + H]^+^: 277.1341, found: 277.1344.

#### 1-Fluoro-10-methoxy-6-methyl-6*H*-indolo[2,3-*b*]quinoline (2w)

Yellow solid; 53.2 mg, 95% yield; mp 197–199 °C. ^1^H NMR (400 MHz, CDCl_3_) *δ* 9.10 (s, 1H), 7.89 (d, *J* = 8.6 Hz, 1H), 7.61–7.56 (m, 1H), 7.54–7.50 (m, 1H), 7.13–7.08 (m, 1H), 7.03 (d, *J* = 8.0 Hz, 1H), 6.79 (d, *J* = 8.2 Hz, 1H), 4.13 (s, 3H), 3.96 (s, 3H). ^13^C NMR (100 MHz, CDCl_3_) *δ* 160.51, 158.00, 157.13, 152.74, 147.17, 144.40, 129.57, 127.82 (d, *J* = 9.7 Hz), 123.33 (d, *J* = 3.6 Hz), 122.78 (d, *J* = 5.8 Hz), 117.55, 115.30 (d, *J* = 16.5 Hz), 109.17, 106.53 (d, *J* = 19.8 Hz), 101.97 (d, *J* = 18.9 Hz), 55.78, 28.11. IR (KBr) 2927.76, 2853.43, 1604.12, 1504.89, 1474.21, 1421.40, 1399.48, 1346.17, 725.34 cm^−1^; HRMS (ESI) calcd for C_17_H_14_FN_2_O [M + H]^+^: 281.1090, found: 281.1087.

#### 10-Methoxy-6,11-dimethyl-6*H*-indolo[2,3-*b*]quinoline (2x)

Yellow solid; 38.6 mg, 70% yield; mp 128–130 °C. ^1^H NMR (400 MHz, CDCl_3_) *δ* 8.28 (dd, *J* = 8.5, 1.0 Hz, 1H), 8.08 (dd, *J* = 8.4, 0.8 Hz, 1H), 7.72–7.66 (m, 1H), 7.54–7.43 (m, 2H), 7.03 (dd, *J* = 8.0, 0.5 Hz, 1H), 6.78 (d, *J* = 8.1 Hz, 1H), 4.07 (s, 3H), 3.95 (s, 3H), 3.42 (s, 3H). ^13^C NMR (100 MHz, CDCl_3_) *δ* 155.49, 152.08, 145.99, 144.63, 139.96, 128.96, 128.48, 127.72, 125.02, 124.88, 122.52, 117.08, 110.01, 102.41, 101.87, 55.51, 28.00, 17.71. IR (KBr) 2926.82, 2854.13, 1608.62, 1589.36, 1386.14, 1362.91, 1316.44, 741.81 cm^−1^; HRMS (ESI) calcd for C_18_H_17_N_2_O [M + H]^+^: 277.1341, found: 277.1345.

## Conflicts of interest

There are no conflicts to declare.

## Supplementary Material

RA-011-D1RA01894A-s001
