# Supplementary material for: PhI(OAc)2-mediated intramolecular oxidative C–N coupling and detosylative aromatization: an access to indolo[2,3-b]quinolines
Source: RSC Adv. 2021 May 10;11(28):17206–11. doi: 10.1039/d1ra01894a (PMC9032537; doi:10.1039/d1ra01894a)
Supplement: RA-011-D1RA01894A-s001 [file RA-011-D1RA01894A-s001.pdf]

Electronic Supplementary Material (ESI) for RSC Advances.
This journal is © The Royal Society of Chemistry 2021

Supporting Information

$PhI(OAc)_2$-Mediated Intramolecular Oxidative C-N Coupling and Detosylative Aromatization: an Access to Indolo[2,3-*b*]quinolines

Quan-Bing Wang †, Shi Tang † *, Ying-Jie Wang, Yue Yuan, Tieqiao Chen and Ai-Qun Jia*

E-mail: tangshi705@163.com

E-mail: ajia@hainanu.edu.cn

Contents

I. Preparation of Starting Materials 1

II. Optimization of the Reaction Conditions 2

III. Copies of ^{1}H and ^{13}C NMR Spectra 5

I. Preparation of Starting Materials

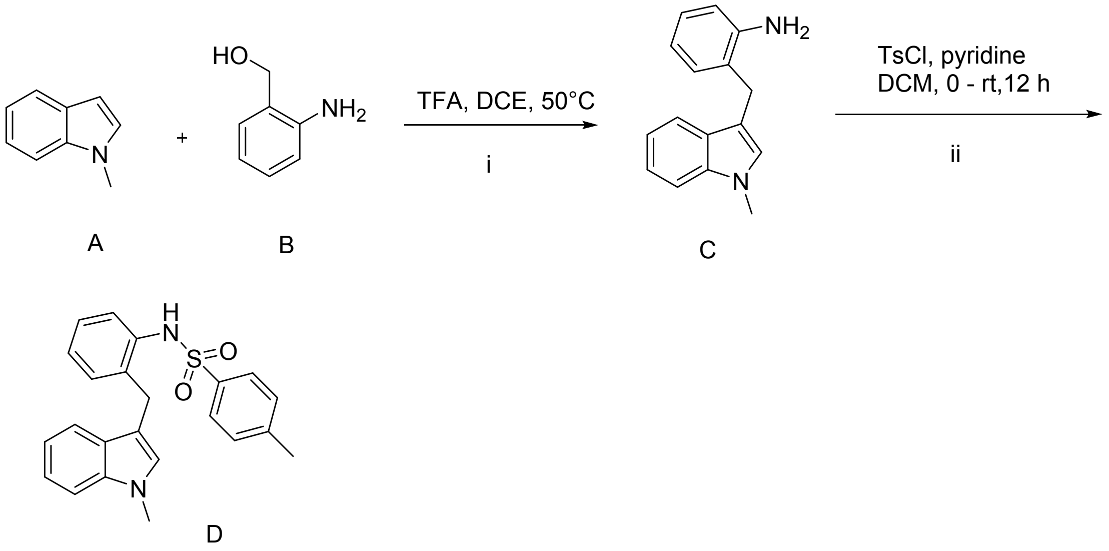

(i) To a solution of A (1.0 equiv) and (2-aminophenyl)-methanol B (2.0 equiv) in DCE was added TFA (30 mol%) at room temperature. The resulting solution was stirred at 50 ^{o}C for 12 h. After the reaction was completed (monitored by TLC), the reaction was quenched with saturated aqueous $NaHCO_3$, then diluted with DCM and washed with brine. The organic phase was dried over Na_2SO_4, filtered and concentrated. The residue was purified by flash chromatography (petroleum ether/ethyl acetate = 5/1) to afford the desired product C.

(ii) The intermediate C (1.0 equiv) was dissolved in dry DCM and the solution was cooled to 0 ^{o}C. Pyridine (1.3 equiv) and TsCl (1.2 equiv) were then added dropwise. The reaction mixture was warmed to room temperature and stirred at this temperature for 12 h until TLC analysis showed a complete consumption of the starting material. HCl (1 N) was added and the organic layer was washed with water. The aqueous layer was extracted with DCM. The combined organic layer was dried over anhydrous Na_2SO_4, filtered and concentrated. The residue was purified by flash chromatography (petroleum ether/ethyl acetate = 5/1) to afford the desired product D.

II. Optimization of the Reaction Conditions[a]

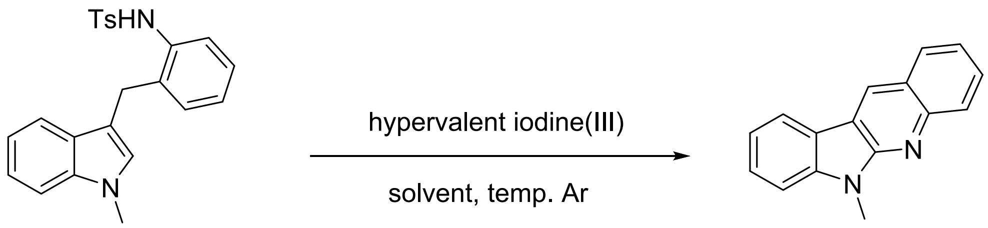

Entry	Oxidant	Solvent	Temp. (℃)	Additive	Yield (%)[b]
1	PIDA	DCM	rt	-	10
2	PIFA	DCM	rt	-	trace
3	PIDA	DCE	rt	-	22
4	PIDA	THF	rt	-	28
5	PIDA	dioxane	rt	-	19
6	PIDA	$PhCF_3$	rt	-	16
7	PIDA	TFE	rt	-	51
8	PIDA	HFIP	rt	-	67
9	PIDA	HFIP	0	-	15
10[c]	PIDA	HFIP	rt	-	43
11	PIDA	HFIP	0 - rt	-	82
12[d]	PIDA	HFIP	0 - rt	CS_2CO_3	73

[a]Reaction condition: 1a (0.2 mmol), Oxidant (0.24 mmol), in Ar, solvent (2 mL) was added at rt and stirred for 12 h. [b]Isolated yield in parentheses [c] PIDA was 4.0 mmol. [d]Cs_2CO_3 is 0.24 mmol.

Screening of protecting group R [a]

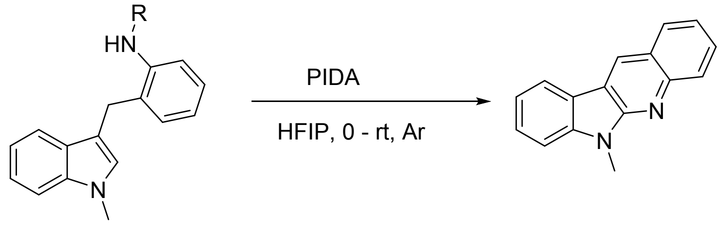

Entry	Oxidant	Solvent	Temp. (℃)	R	Yield (%)[b]
13	PIDA	HFIP	0 - rt	benzenesulfonyl	73
14	PIDA	HFIP	0 - rt	4-chlorobenzenesulfonyl	86
15	PIDA	HFIP	0 - rt	4-fluorobenzenesulfonyl	75
16	PIDA	HFIP	0 - rt	methylsulfonyl	74
17	PIDA	HFIP	0 – rt	acetyl	trace

[a]Reaction condition: 1 (0.2 mmol), PIDA (0.24 mmol), in Ar, HFIP (2 mL) was added at 0 °C, then temperature was increased to rt stirred for 12 h. [b]Isolated yield in parentheses.

Screening of oxidant and solvent [a]

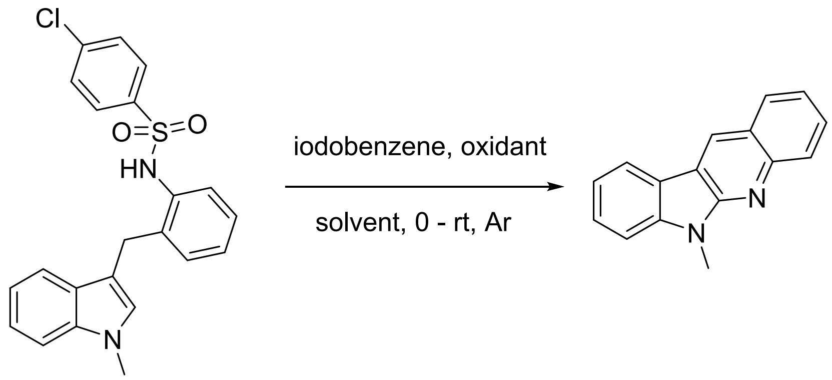

Entry	Oxidant	Solvent	Temp. (℃)	Additive	Yield (%) [b]
18	H_2O_2	DCM	0 - rt	-	--
29	*m*-CPBA	DCM	0 - rt	-	--
20	*m*-CPBA	CF_3CH_2OH	0 - rt	-	23
21	*m*-CPBA	HFIP	0 - rt	-	25

[a]Reaction condition: 1 (0.2 mmol), iodobenzene (0.06 mmol), oxidant (0.24 mmol) in Ar, solvent (2 mL) was added at 0 ℃，then temperature was increased to rt and stirred for 12 h. [b]Isolated yield in parentheses.

III. Copies of ^{1}H and ^{13}C NMR Spectra

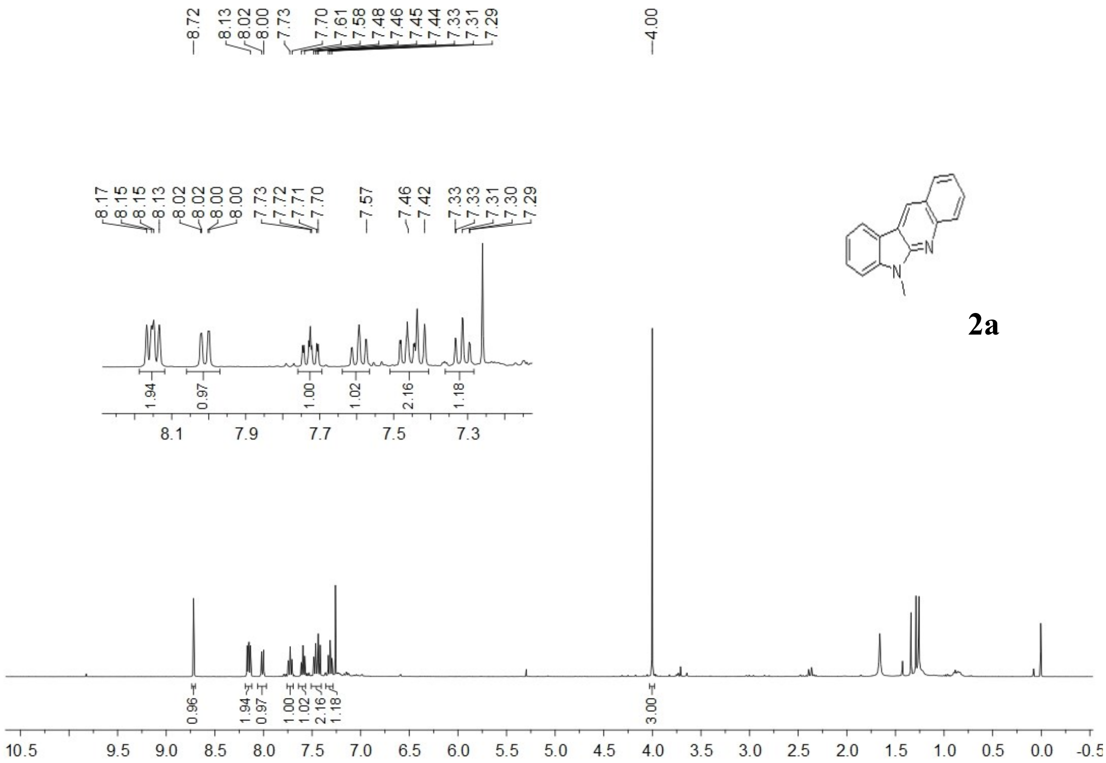

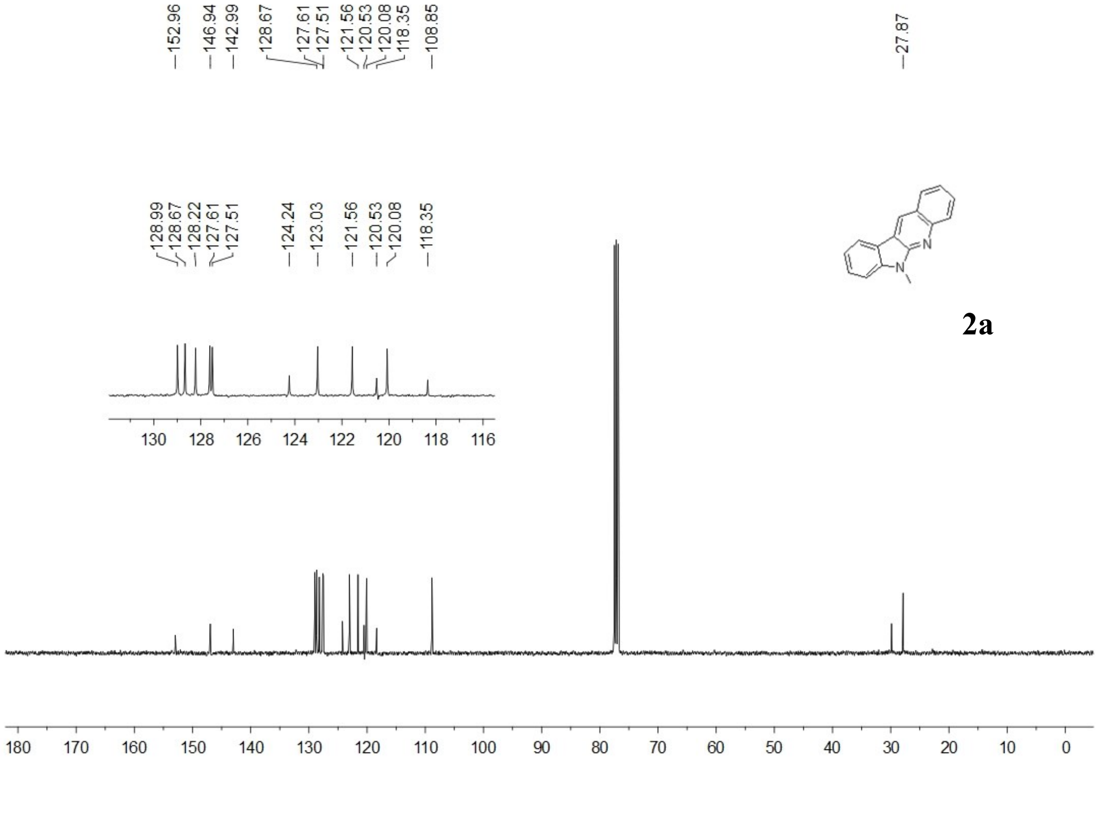

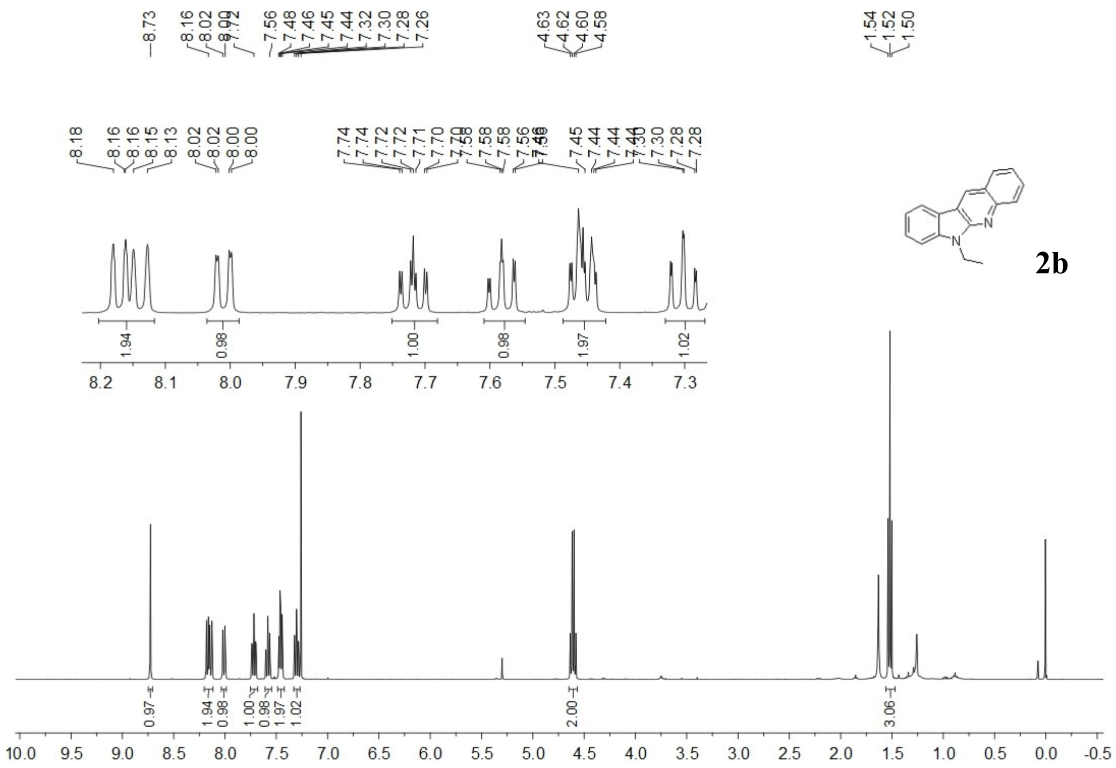
8.73
8.16 8.02 8.00 7.72
7.56 7.48 7.46 7.45 7.44 7.32 7.30 7.28 7.26
4.63 4.62 4.60 4.58
1.54 1.52 1.50
8.18 8.16 8.16 8.15 8.13 8.02 8.02 8.00 8.00
7.74 7.74 7.72 7.72 7.71 7.70 7.58 7.58 7.56 7.45 7.44 7.44 7.30 7.28 7.28
1.94 0.98 1.00 0.98 1.97 1.02
2b
0.97 1.94 0.98 1.00 0.98 1.97 1.02 2.00 3.06

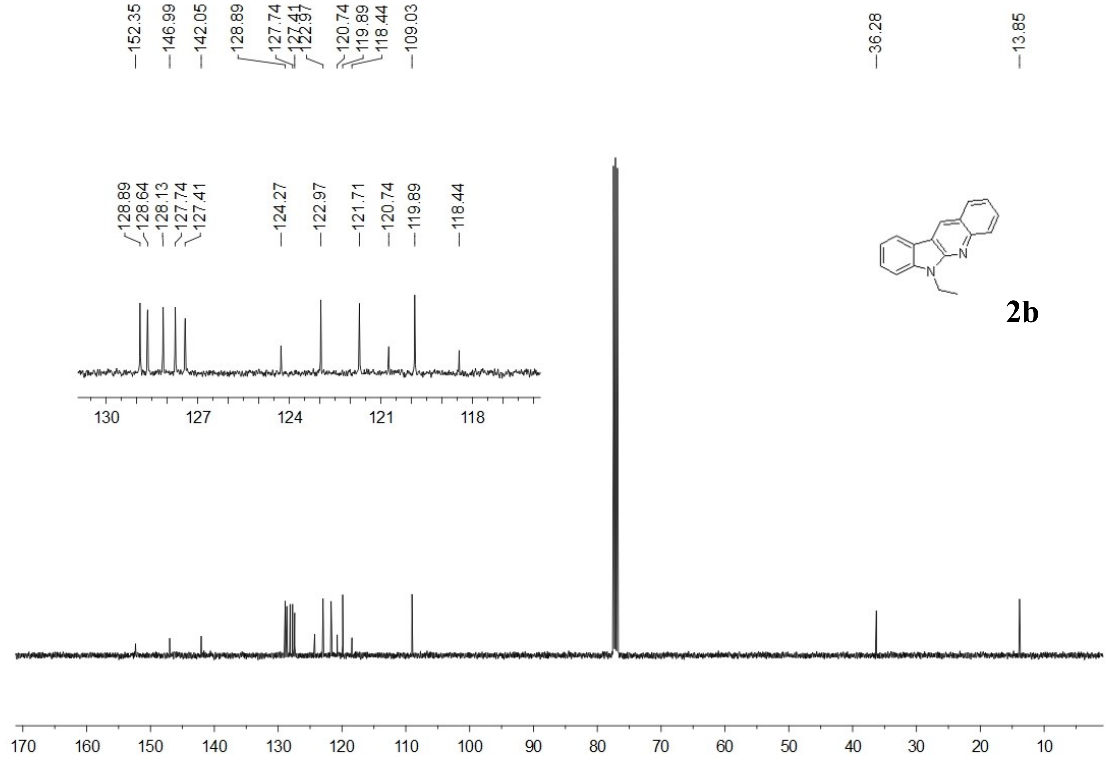
152.35 146.99 142.05 128.89 127.74 127.41 122.97 120.74 119.89 118.44 109.03
36.28 13.85
128.89 128.64 128.13 127.74 127.41 124.27 122.97 121.71 120.74 119.89 118.44
2b

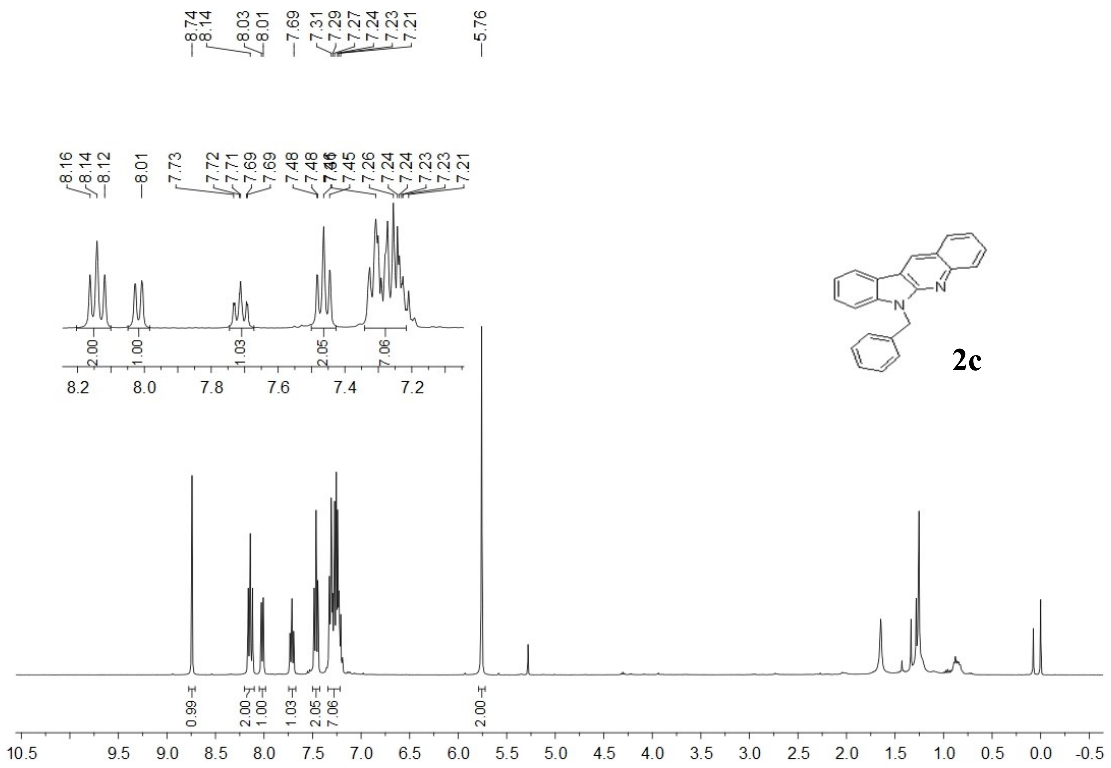
8.74
8.14
8.03
8.01
7.69
7.31
7.29
7.27
7.24
7.23
7.21
5.76
8.16
8.14
8.12
8.01
7.73
7.72
7.71
7.69
7.69
7.48
7.48
7.45
7.26
7.24
7.24
7.23
7.23
7.21
2.00
1.00
1.03
2.05
7.06
8.2 8.0 7.8 7.6 7.4 7.2
N
2c
0.99
2.00
1.00
1.03
2.05
7.06
2.00
10.5 9.5 9.0 8.5 8.0 7.5 7.0 6.5 6.0 5.5 5.0 4.5 4.0 3.5 3.0 2.5 2.0 1.5 1.0 0.5 0.0 -0.5

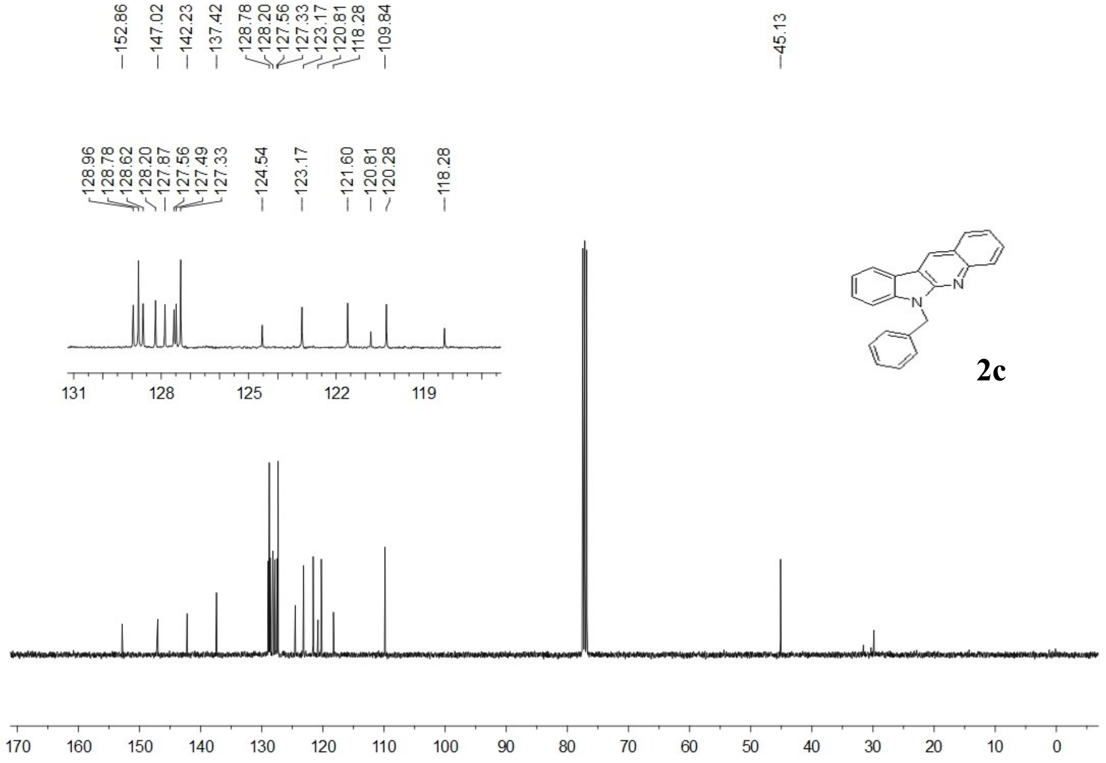
152.86
147.02
142.23
137.42
128.78
128.20
127.56
127.33
123.17
120.81
118.28
109.84
45.13
128.96
128.78
128.62
128.20
127.87
127.56
127.49
127.33
124.54
123.17
121.60
120.81
120.28
118.28
131 128 125 122 119
N
2c
170 160 150 140 130 120 110 100 90 80 70 60 50 40 30 20 10 0

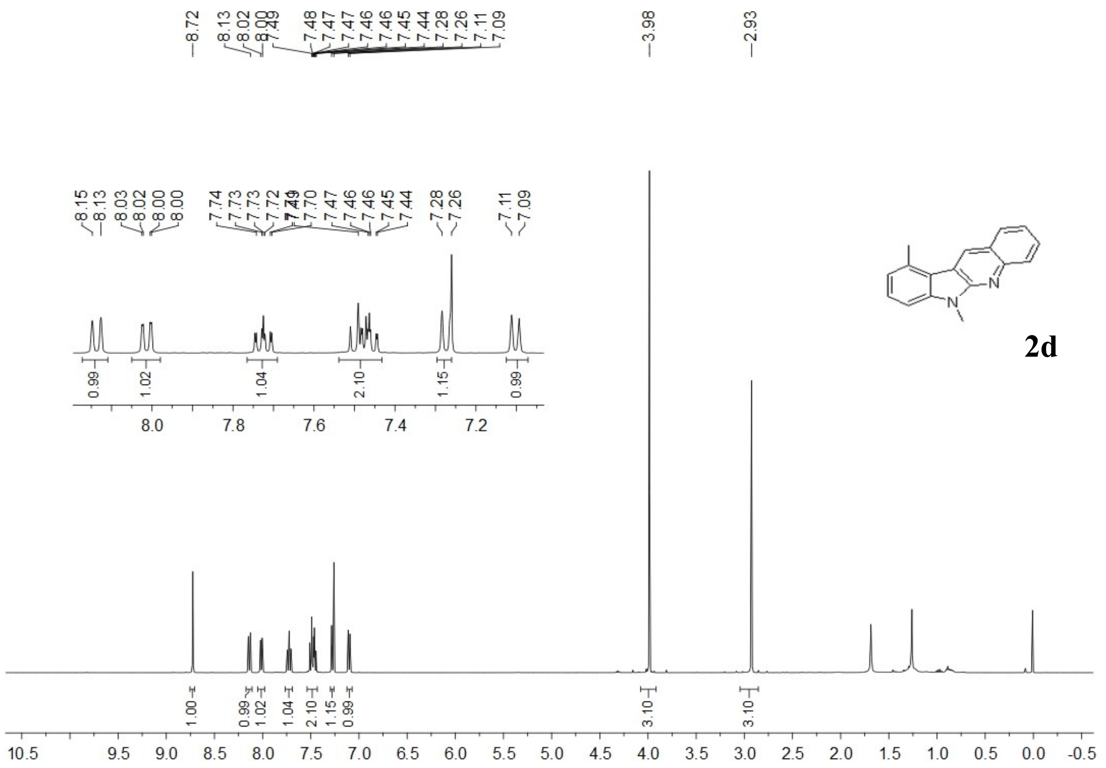
8.72
8.13
8.02
8.00
7.49
7.48
7.47
7.47
7.46
7.46
7.45
7.44
7.28
7.26
7.11
7.09
3.98
2.93
8.15
8.13
8.03
8.02
8.00
8.00
7.74
7.73
7.73
7.72
7.70
7.47
7.46
7.46
7.45
7.44
7.28
7.26
7.11
7.09
0.99
1.02
1.04
2.10
1.15
0.99
8.0
7.8
7.6
7.4
7.2
2d
1.00
3.10
3.10
10.5
9.5
9.0
8.5
8.0
7.5
7.0
6.5
6.0
5.5
5.0
4.5
4.0
3.5
3.0
2.5
2.0
1.5
1.0
0.5
0.0
-0.5

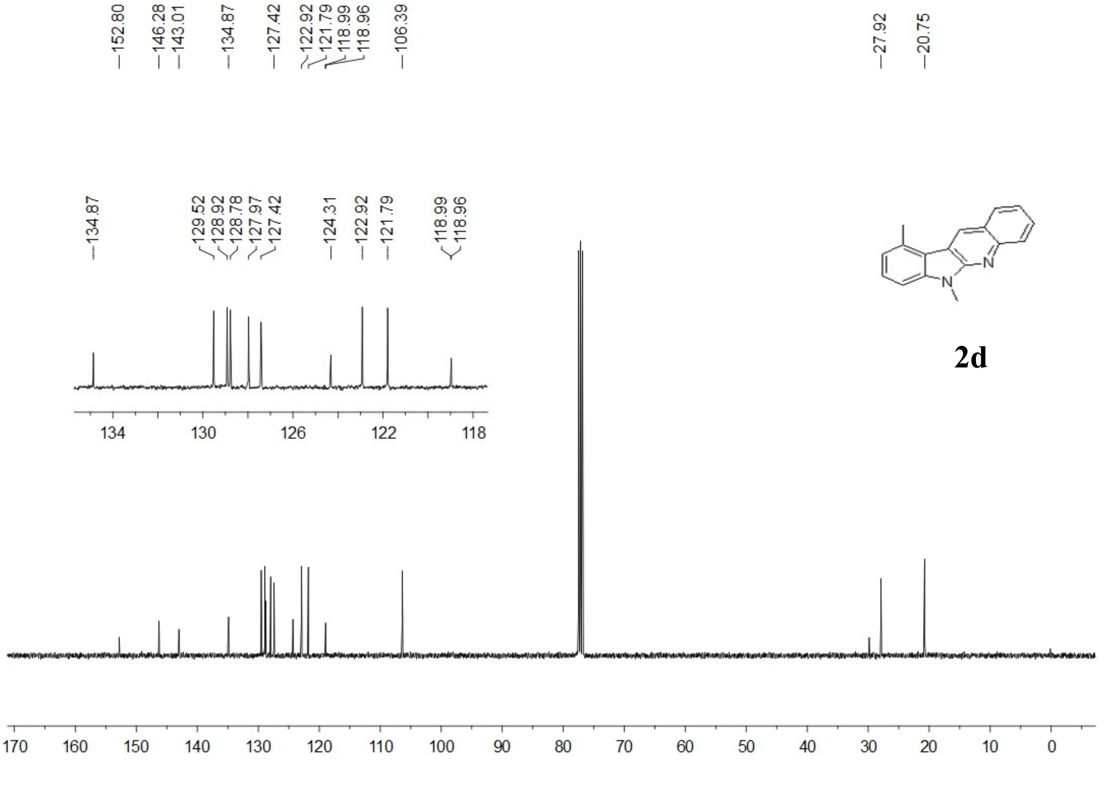
152.80
146.28
143.01
134.87
127.42
122.92
121.79
118.99
118.96
106.39
27.92
20.75
134.87
129.52
128.92
128.78
127.97
127.42
124.31
122.92
121.79
118.99
118.96
134
130
126
122
118
2d
170
160
150
140
130
120
110
100
90
80
70
60
50
40
30
20
10
0

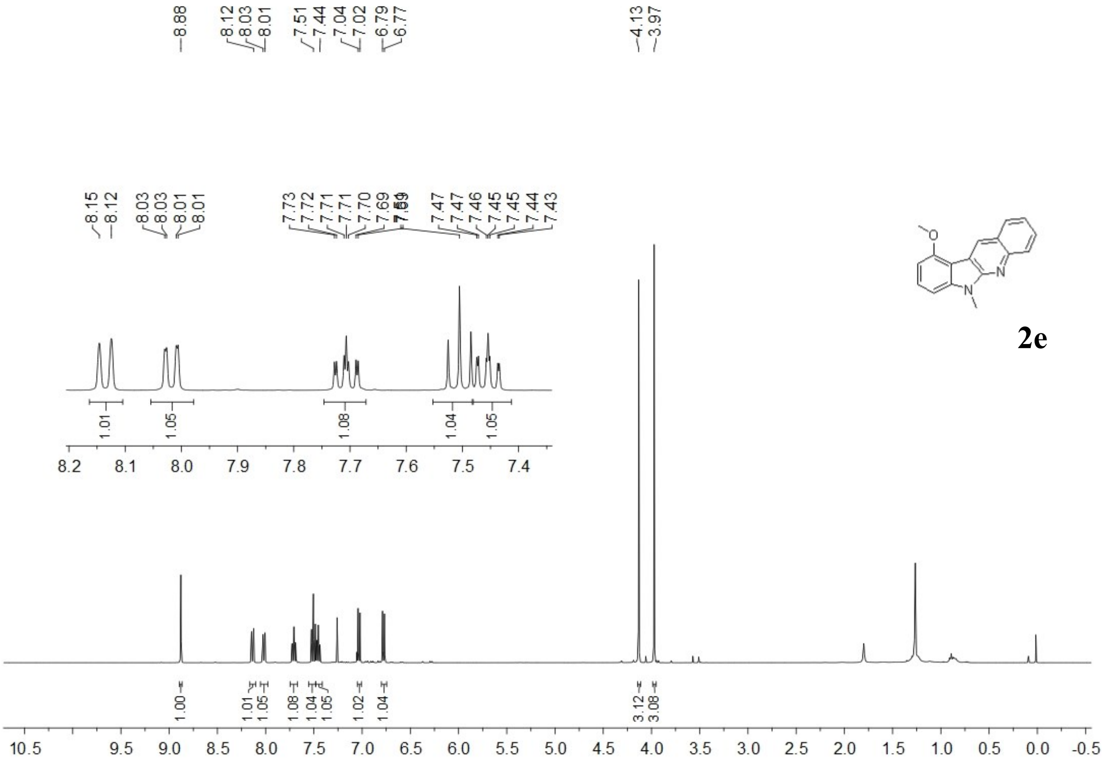
2e

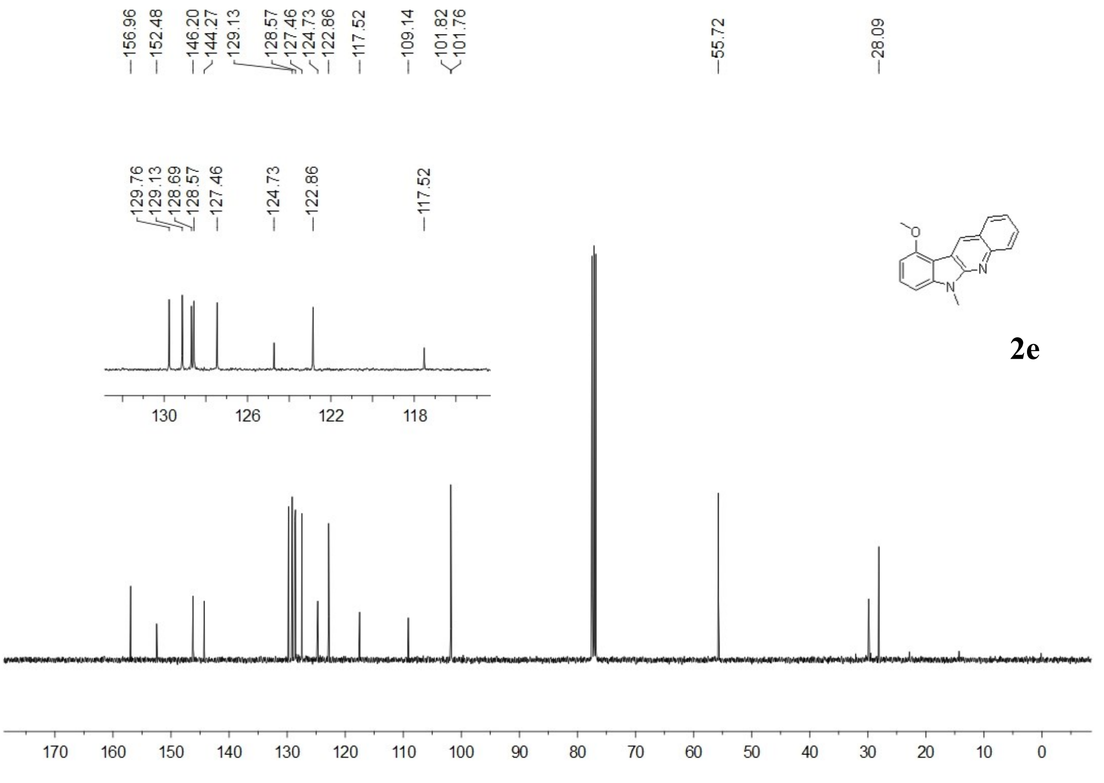
2e

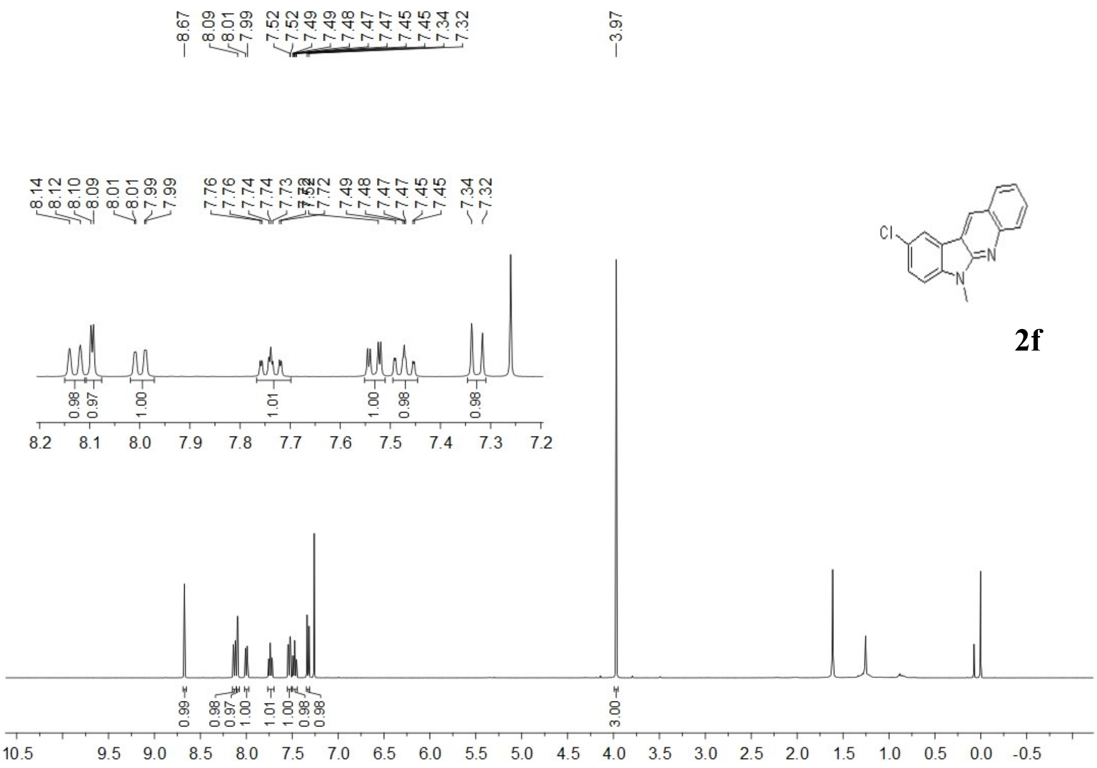
8.67
8.09
8.01
7.99
7.52
7.52
7.49
7.49
7.48
7.47
7.47
7.45
7.45
7.34
7.32
3.97
8.14
8.12
8.10
8.09
8.01
7.99
7.99
7.76
7.76
7.74
7.74
7.73
7.72
7.49
7.48
7.47
7.47
7.45
7.45
7.34
7.32
Cl
N
N
2f
0.98
0.97
1.00
1.01
1.00
0.98
0.98
0.99
3.00

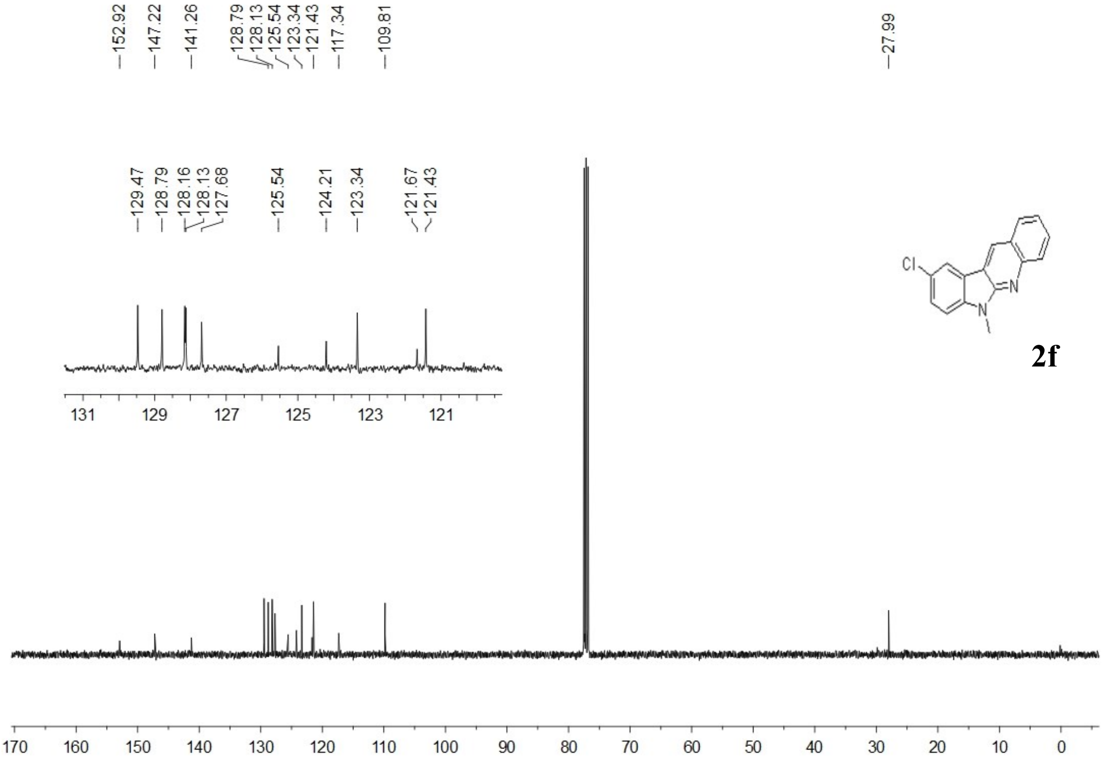
152.92
147.22
141.26
128.79
128.13
125.54
123.34
121.43
117.34
109.81
27.99
129.47
128.79
128.16
128.13
127.68
125.54
124.21
123.34
121.67
121.43
Cl
N
N
2f

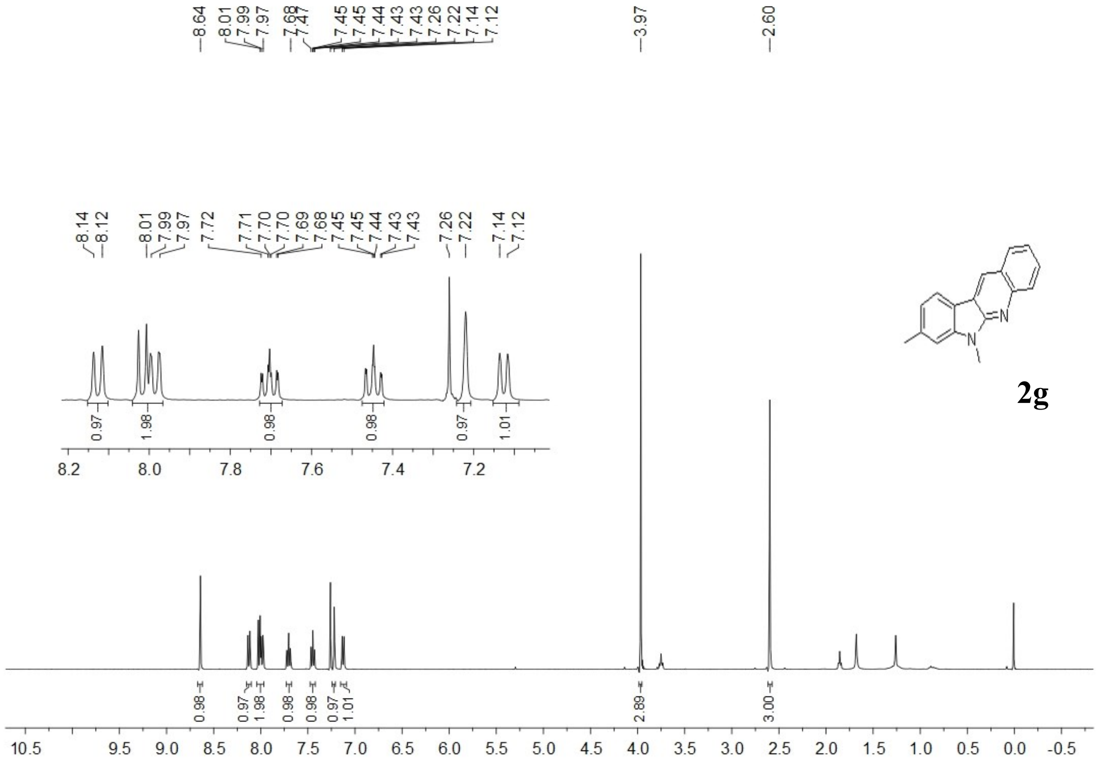

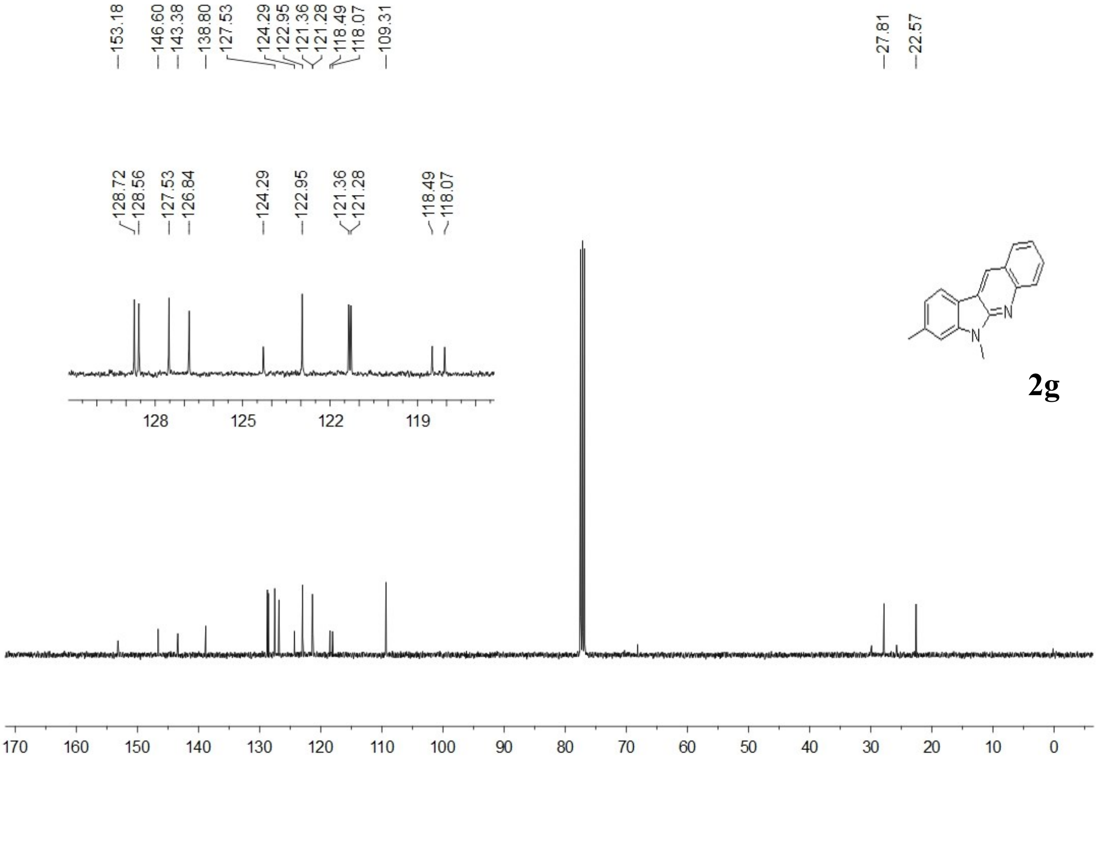

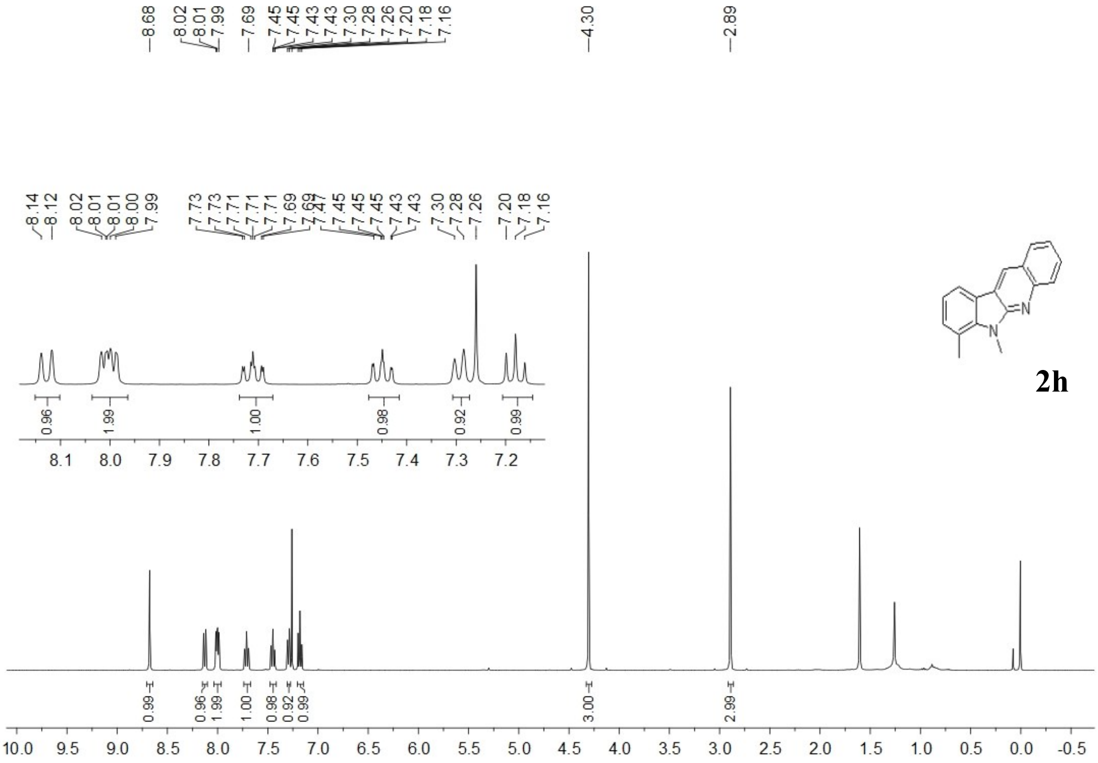
8.68
8.02
8.01
7.99
7.69
7.45
7.45
7.43
7.43
7.30
7.28
7.26
7.20
7.18
7.16
4.30
2.89
8.14
8.12
8.02
8.01
8.01
8.00
7.99
7.73
7.73
7.71
7.71
7.71
7.69
7.69
7.47
7.45
7.45
7.45
7.43
7.43
7.30
7.28
7.26
7.20
7.18
7.16
0.96
1.99
1.00
0.98
0.92
0.99
8.1 8.0 7.9 7.8 7.7 7.6 7.5 7.4 7.3 7.2
N
2h
0.99
0.96
1.99
1.00
0.98
0.92
0.99
3.00
2.99
10.0 9.5 9.0 8.5 8.0 7.5 7.0 6.5 6.0 5.5 5.0 4.5 4.0 3.5 3.0 2.5 2.0 1.5 1.0 0.5 0.0 -0.5

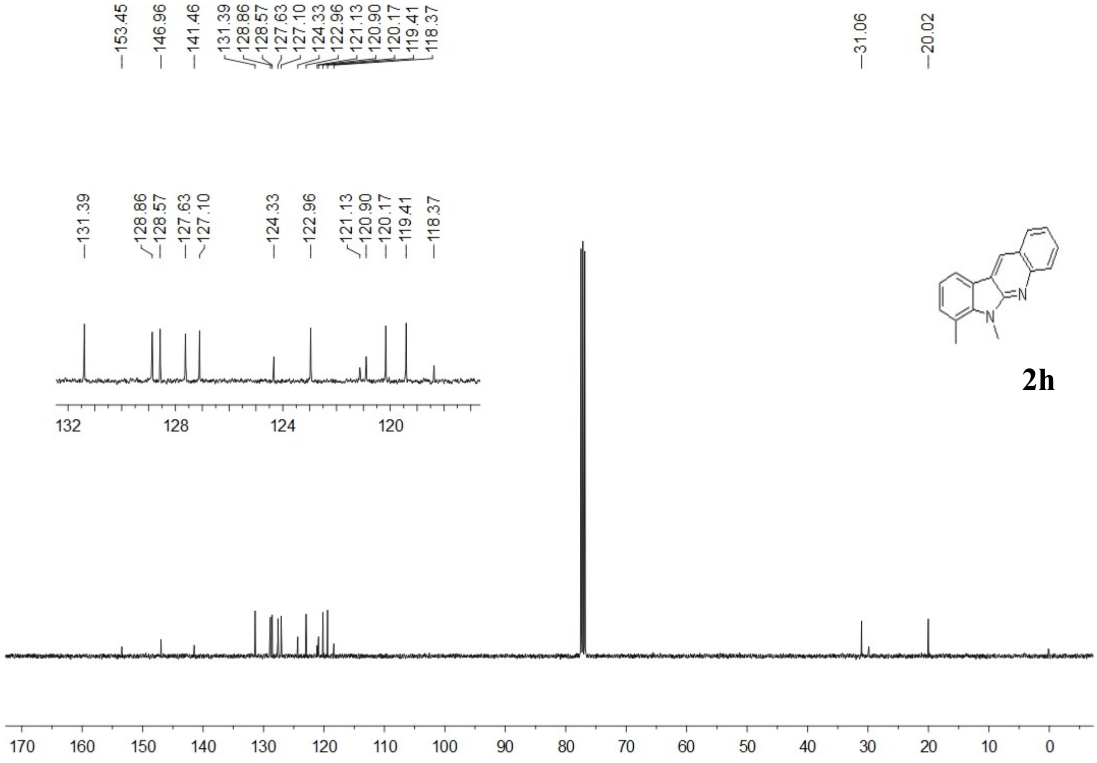
153.45
146.96
141.46
131.39
128.86
128.57
127.63
127.10
124.33
122.96
121.13
120.90
120.17
119.41
118.37
31.06
20.02
132 128 124 120
N
2h
170 160 150 140 130 120 110 100 90 80 70 60 50 40 30 20 10 0

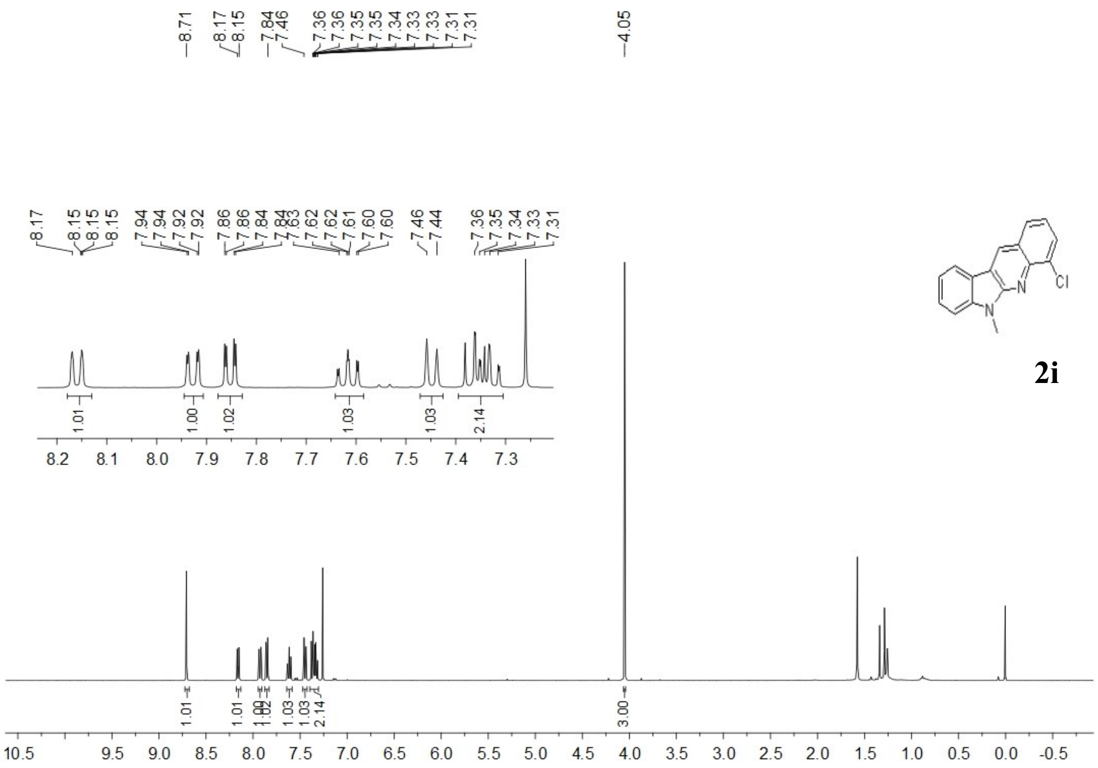
8.71
8.17
8.15
7.84
7.46
7.36
7.36
7.35
7.35
7.34
7.33
7.33
7.31
7.31
4.05
8.17
8.15
8.15
8.15
7.94
7.94
7.92
7.92
7.86
7.86
7.84
7.84
7.63
7.62
7.62
7.61
7.60
7.60
7.46
7.44
7.36
7.35
7.34
7.33
7.31
1.01
1.00
1.02
1.03
1.03
2.14
Cl
N
2i
3.00

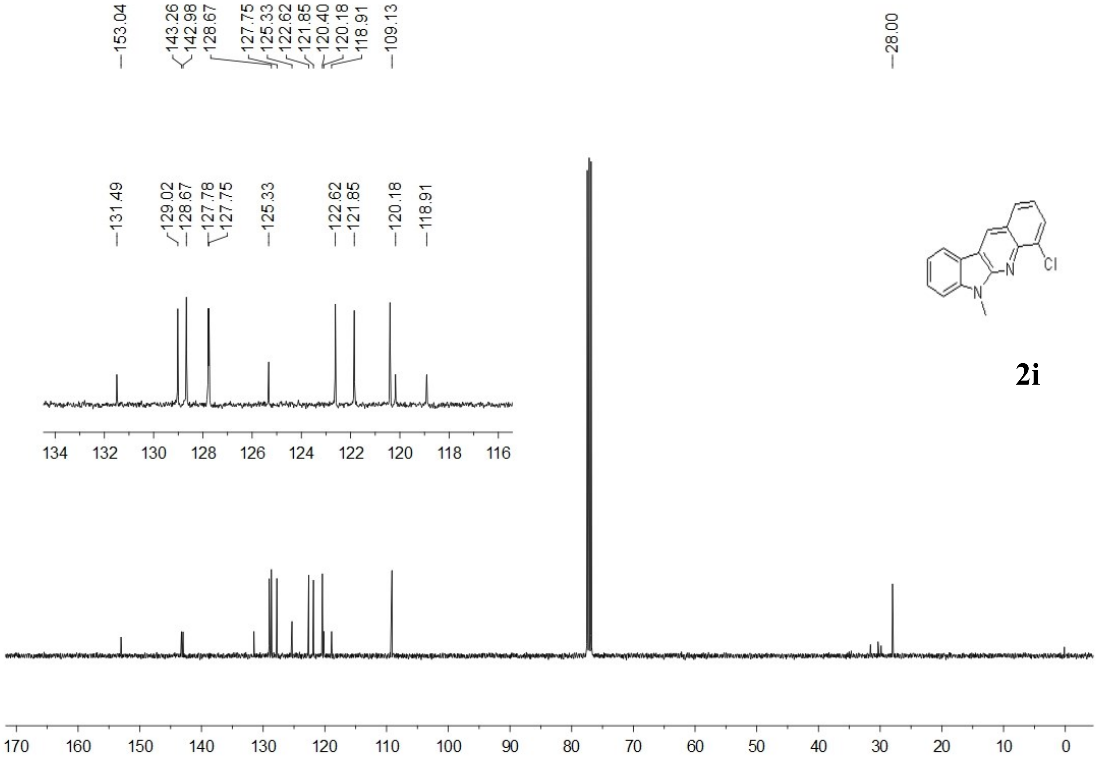
153.04
143.26
142.98
128.67
127.75
125.33
122.62
121.85
120.40
120.18
118.91
109.13
28.00
131.49
129.02
128.67
127.78
127.75
125.33
122.62
121.85
120.18
118.91
Cl
N
2i

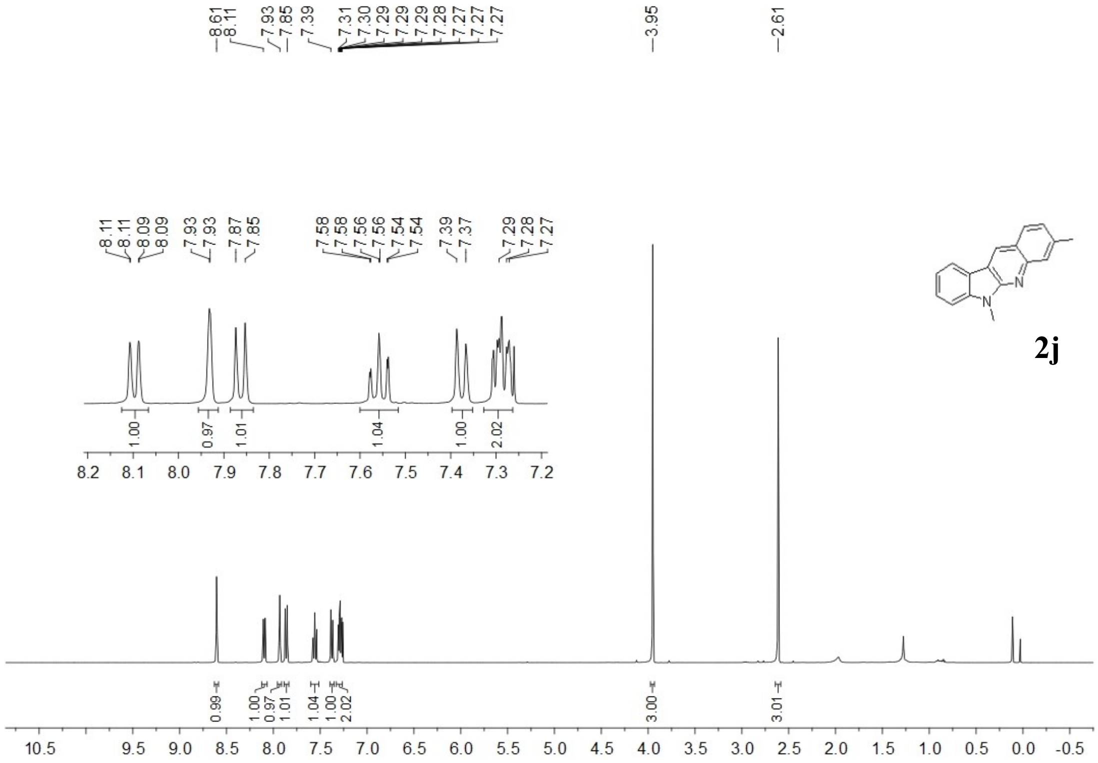
8.61
8.11
7.93
7.85
7.39
7.31
7.30
7.29
7.29
7.29
7.28
7.27
7.27
7.27
3.95
2.61
8.11
8.11
8.09
8.09
7.93
7.93
7.87
7.85
7.58
7.58
7.56
7.56
7.54
7.54
7.39
7.37
7.29
7.28
7.27
1.00
0.97
1.01
1.04
1.00
2.02
0.99
3.00
3.01
2j

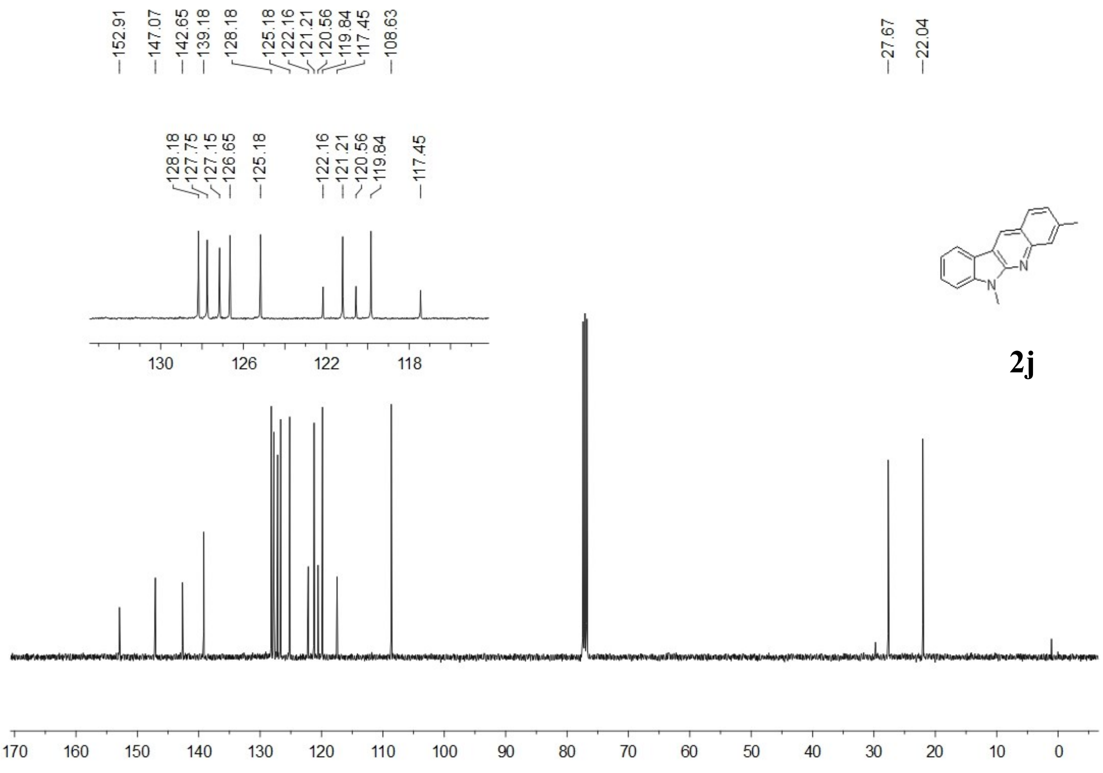
152.91
147.07
142.65
139.18
128.18
125.18
122.16
121.21
120.56
119.84
117.45
108.63
27.67
22.04
128.18
127.75
127.15
126.65
125.18
122.16
121.21
120.56
119.84
117.45
2j

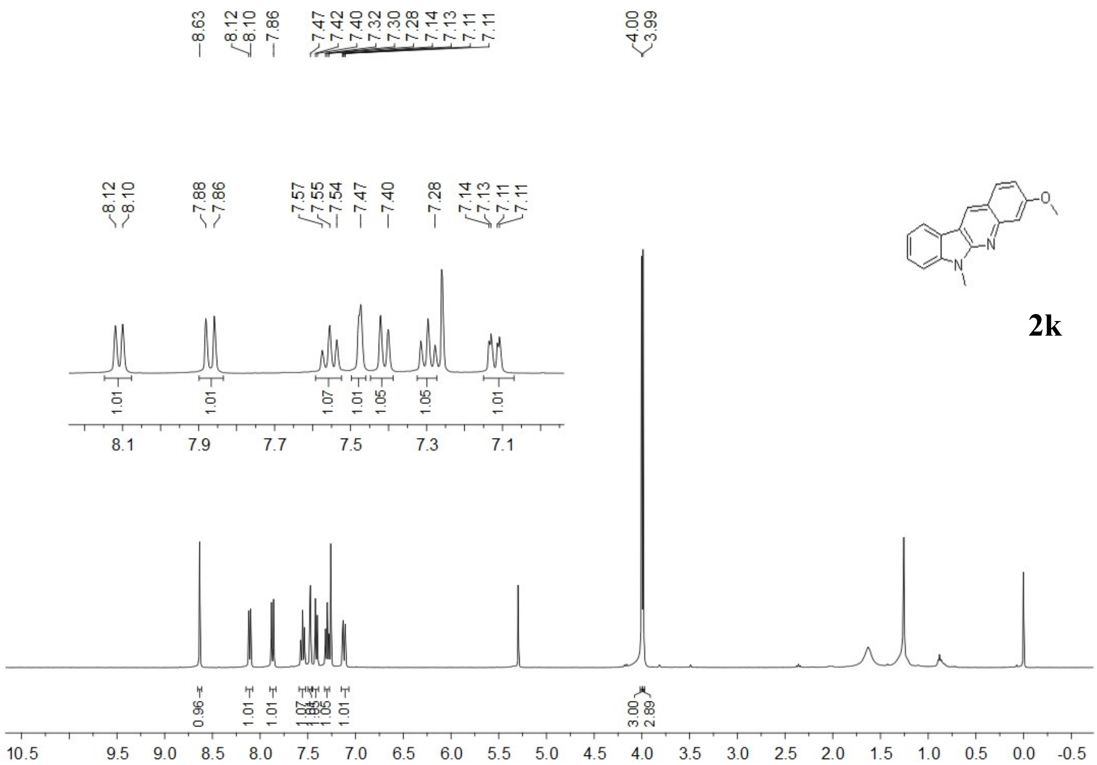

2k

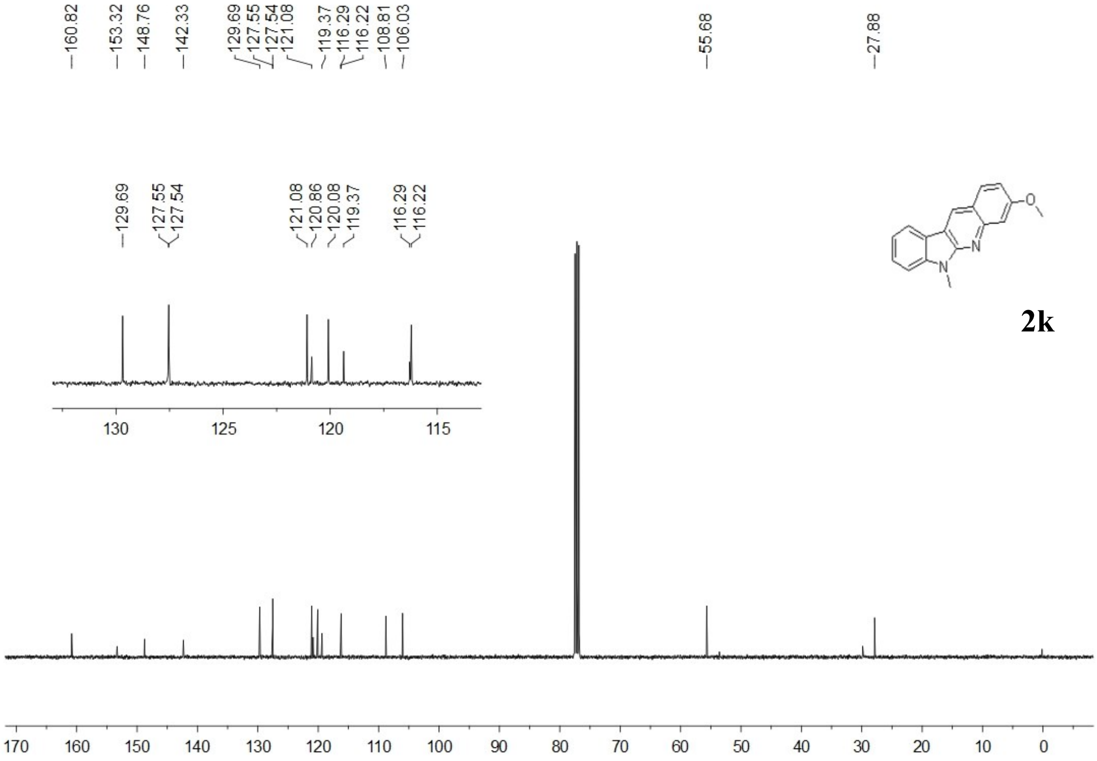

2k

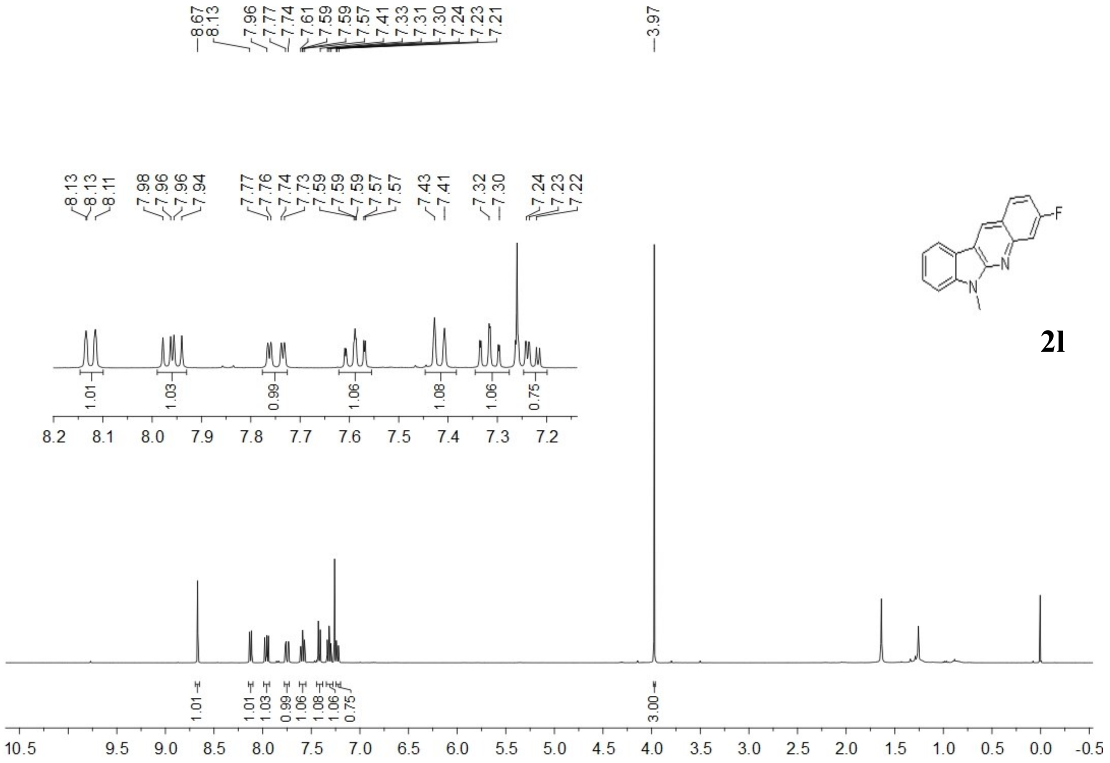
8.67
8.13
7.96
7.77
7.74
7.61
7.59
7.59
7.57
7.41
7.33
7.31
7.30
7.24
7.23
7.21
3.97
8.13
8.13
8.11
7.98
7.96
7.96
7.94
7.77
7.76
7.74
7.73
7.59
7.59
7.59
7.57
7.57
7.43
7.41
7.32
7.30
7.24
7.23
7.22
F
N
N
2l
1.01
1.03
0.99
1.06
1.08
1.06
0.75
3.00

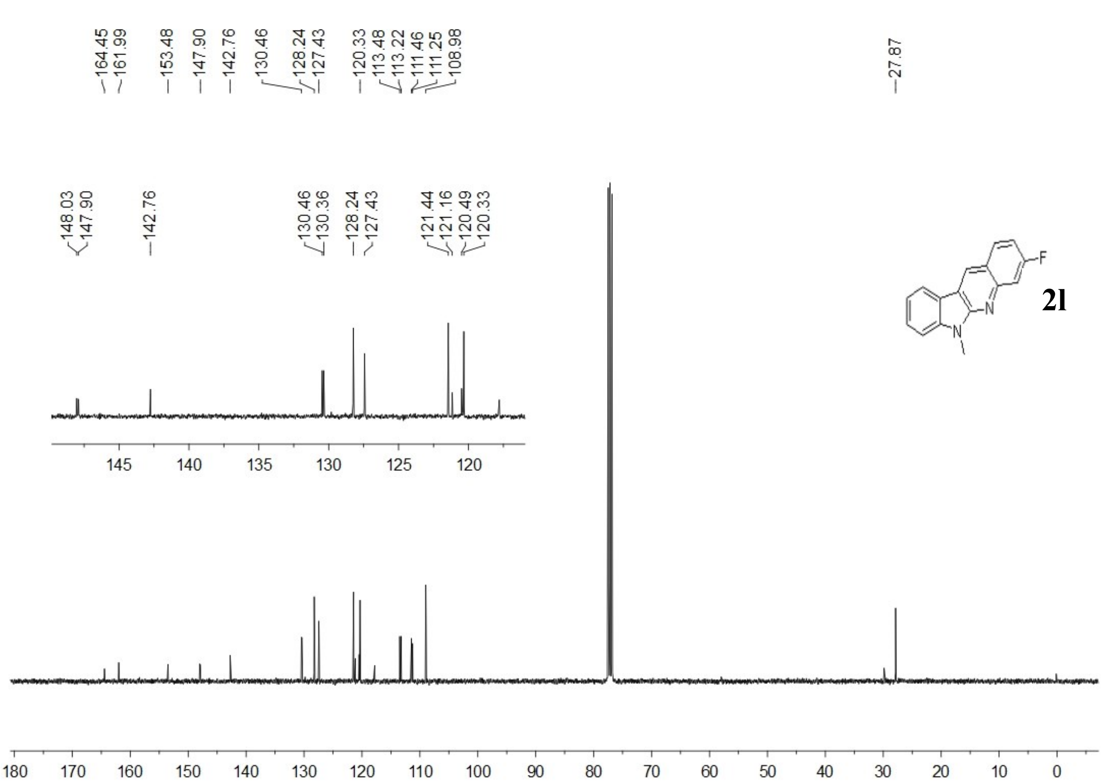
164.45
161.99
153.48
147.90
142.76
130.46
128.24
127.43
120.33
113.48
113.22
111.46
111.25
108.98
27.87
148.03
147.90
142.76
130.46
130.36
128.24
127.43
121.44
121.16
120.49
120.33
F
N
N
2l

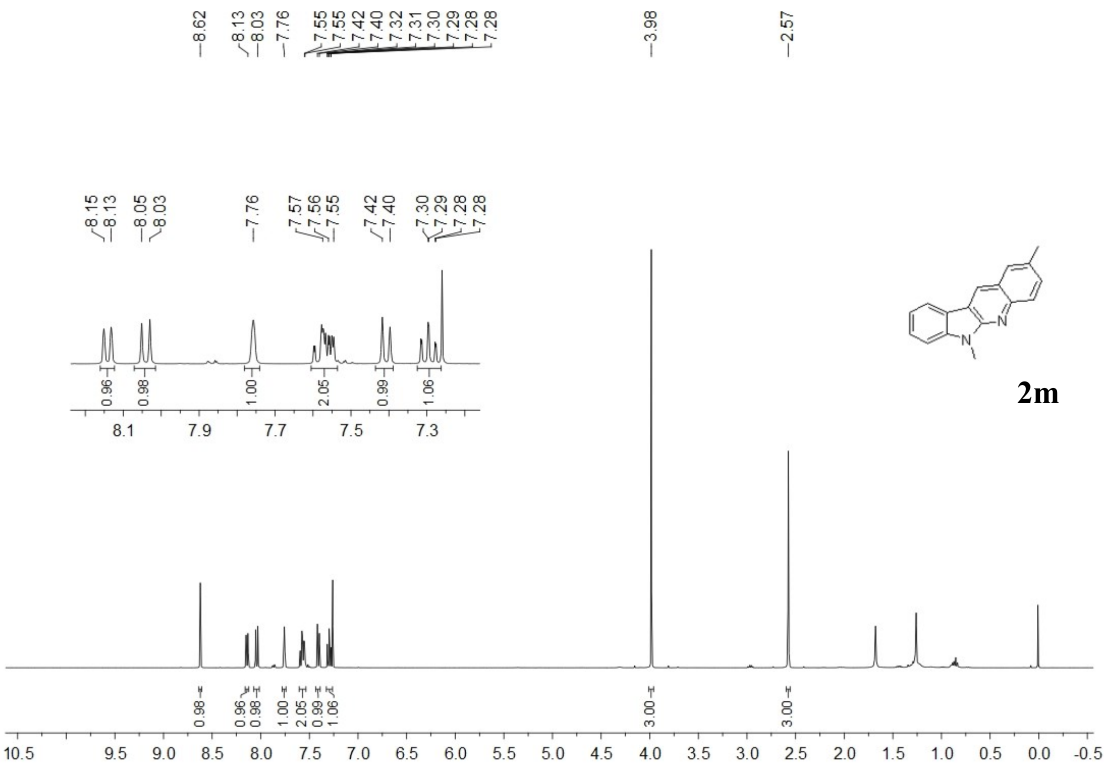
8.62
8.13
8.03
7.76
7.55
7.55
7.42
7.40
7.32
7.31
7.30
7.29
7.28
7.28
3.98
2.57
8.15
8.13
8.05
8.03
7.76
7.57
7.56
7.55
7.42
7.40
7.30
7.29
7.28
7.28
0.96
0.98
1.00
2.05
0.99
1.06
8.1
7.9
7.7
7.5
7.3
2m
0.98
0.96
0.98
1.00
2.05
0.99
1.06
3.00
3.00
10.5
9.5
9.0
8.5
8.0
7.5
7.0
6.5
6.0
5.5
5.0
4.5
4.0
3.5
3.0
2.5
2.0
1.5
1.0
0.5
0.0
-0.5

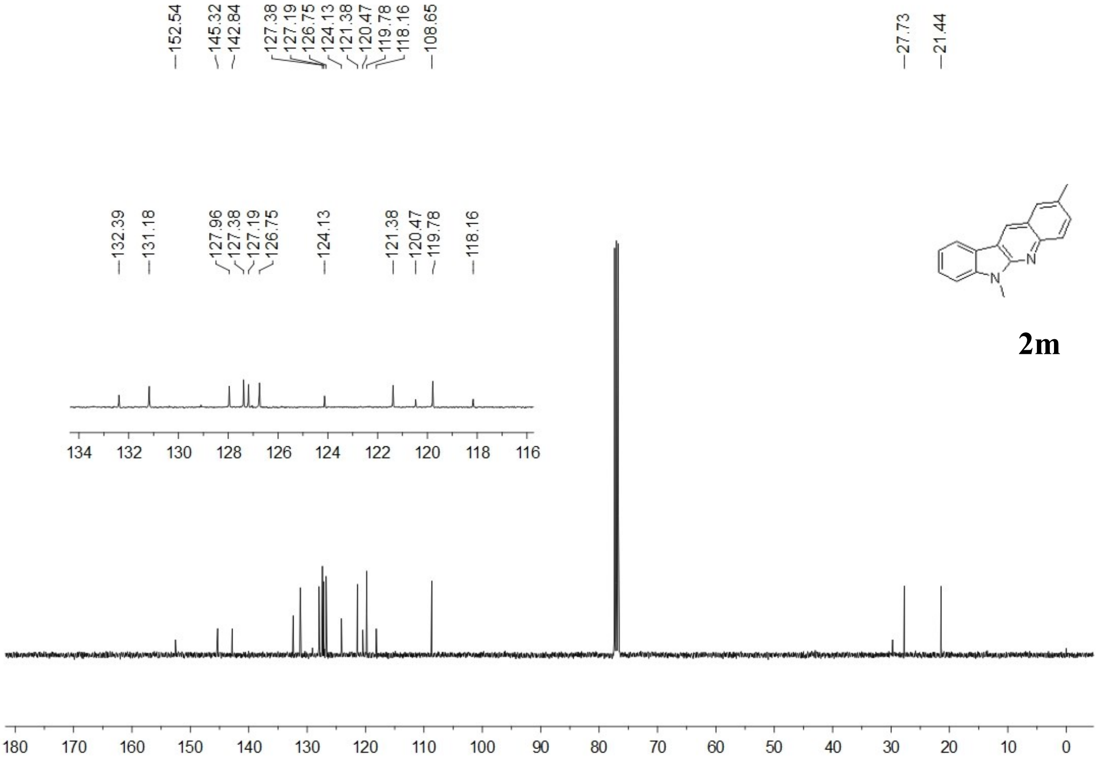
152.54
145.32
142.84
127.38
127.19
126.75
124.13
121.38
120.47
119.78
118.16
108.65
27.73
21.44
132.39
131.18
127.96
127.38
127.19
126.75
124.13
121.38
120.47
119.78
118.16
134
132
130
128
126
124
122
120
118
116
2m
180
170
160
150
140
130
120
110
100
90
80
70
60
50
40
30
20
10
0

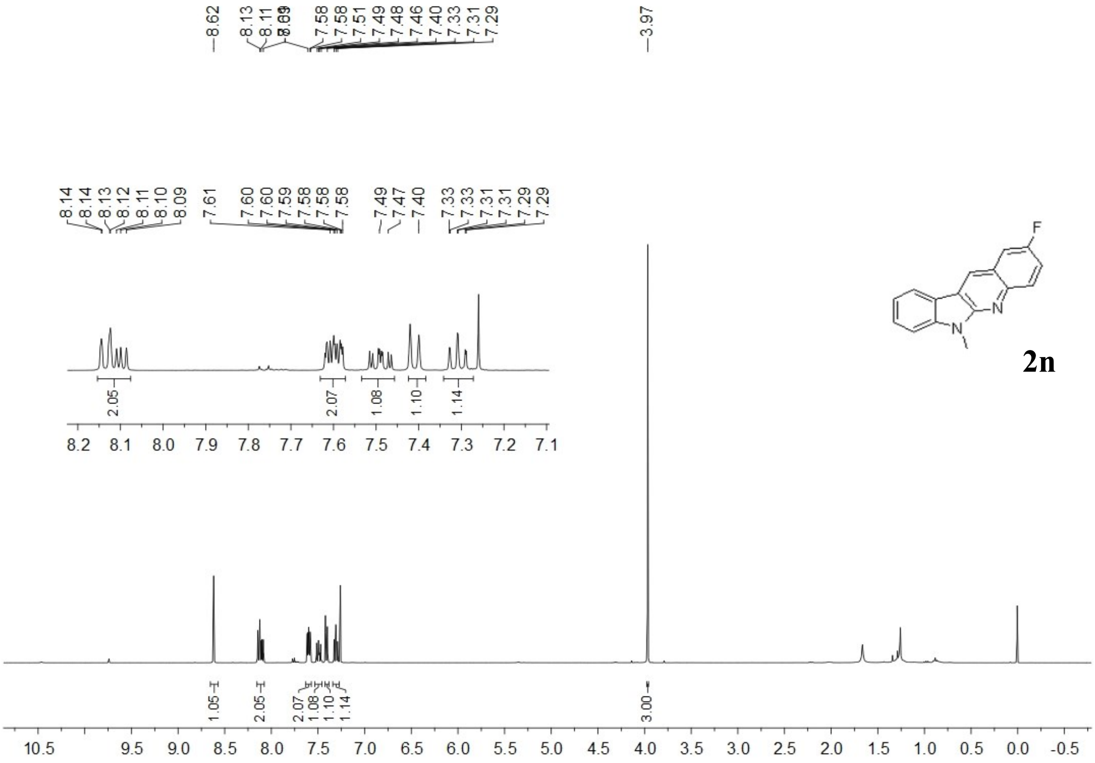
8.62
8.13
8.11
8.09
7.58
7.58
7.51
7.49
7.48
7.46
7.40
7.33
7.31
7.29
3.97
8.14
8.14
8.13
8.12
8.11
8.10
8.09
7.61
7.60
7.60
7.59
7.58
7.58
7.58
7.58
7.49
7.47
7.40
7.33
7.33
7.31
7.31
7.29
7.29
2.05
2.07
1.08
1.10
1.14
1.05
3.00
F
N
2n

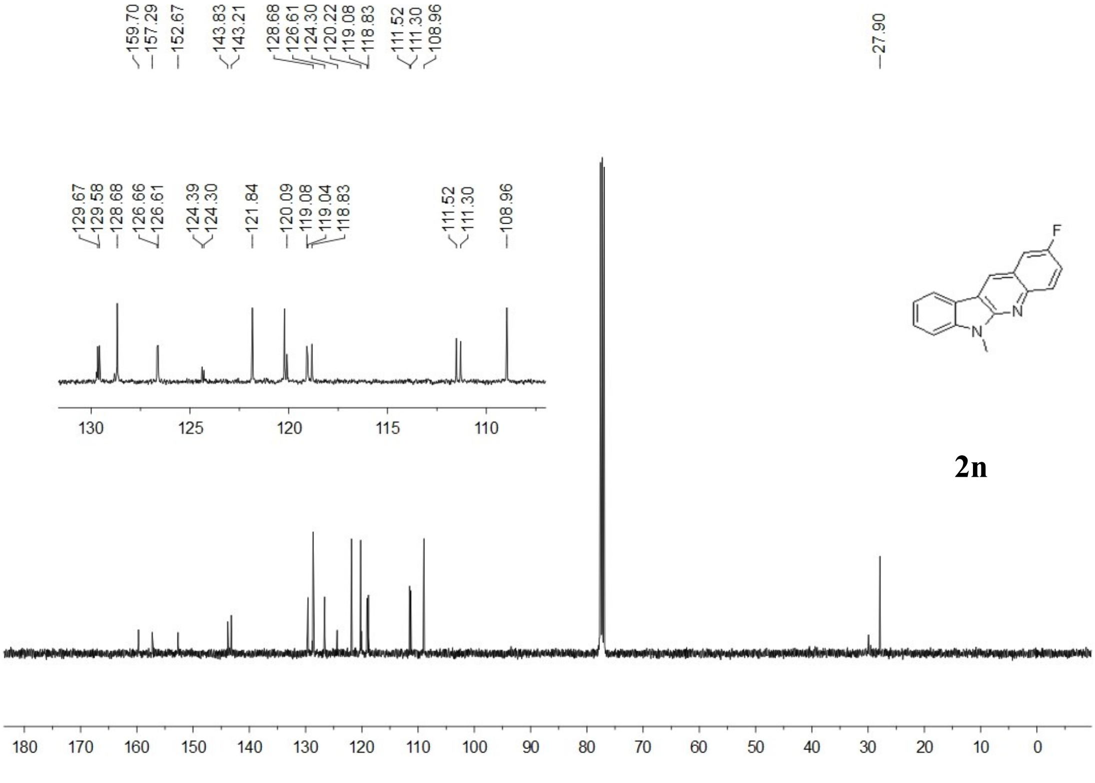
159.70
157.29
152.67
143.83
143.21
128.68
126.61
124.30
120.22
119.08
118.83
111.52
111.30
108.96
27.90
129.67
129.58
128.68
126.66
126.61
124.39
124.30
121.84
120.09
119.08
119.04
118.83
111.52
111.30
108.96
F
N
2n

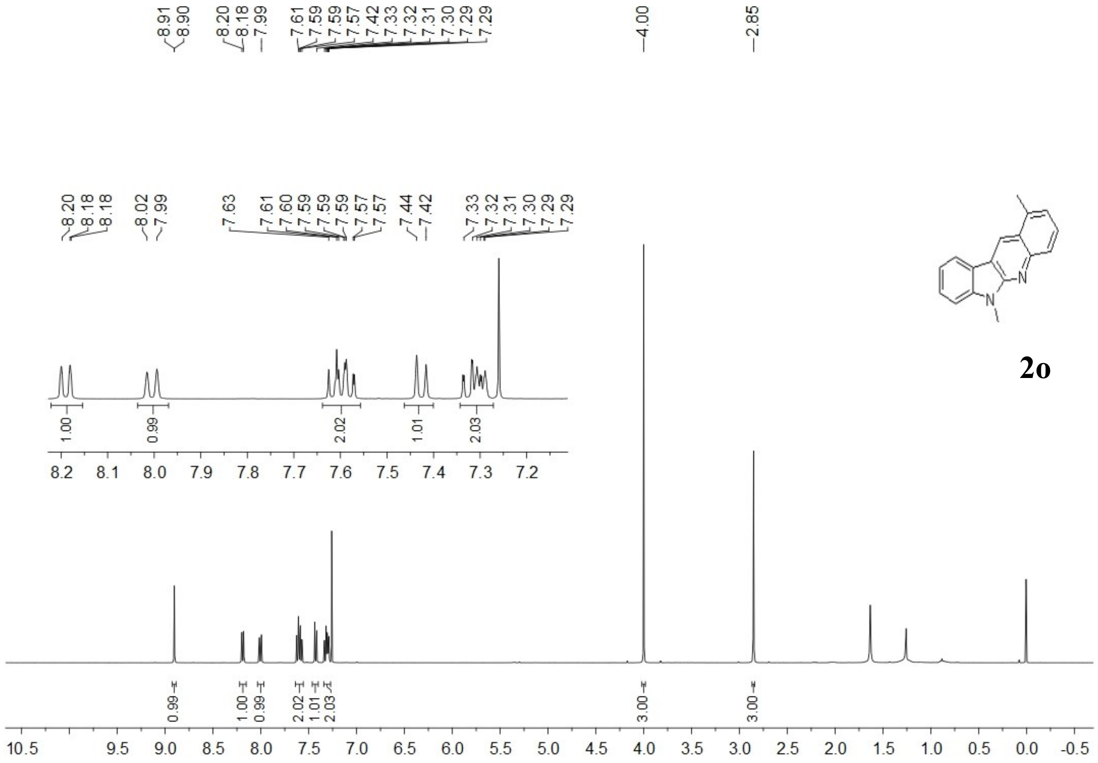
8.91 8.90 8.20 8.18 7.99 7.61 7.59 7.59 7.57 7.42 7.33 7.32 7.31 7.30 7.29 7.29
4.00
2.85
8.20 8.18 8.18 8.02 7.99 7.63 7.61 7.60 7.59 7.59 7.59 7.57 7.57 7.44 7.42 7.33 7.32 7.31 7.30 7.29 7.29
1.00 0.99 2.02 1.01 2.03
8.2 8.1 8.0 7.9 7.8 7.7 7.6 7.5 7.4 7.3 7.2
0.99 1.00 0.99 2.02 1.01 2.03 3.00 3.00
10.5 9.5 9.0 8.5 8.0 7.5 7.0 6.5 6.0 5.5 5.0 4.5 4.0 3.5 3.0 2.5 2.0 1.5 1.0 0.5 0.0 -0.5
N
N

2o

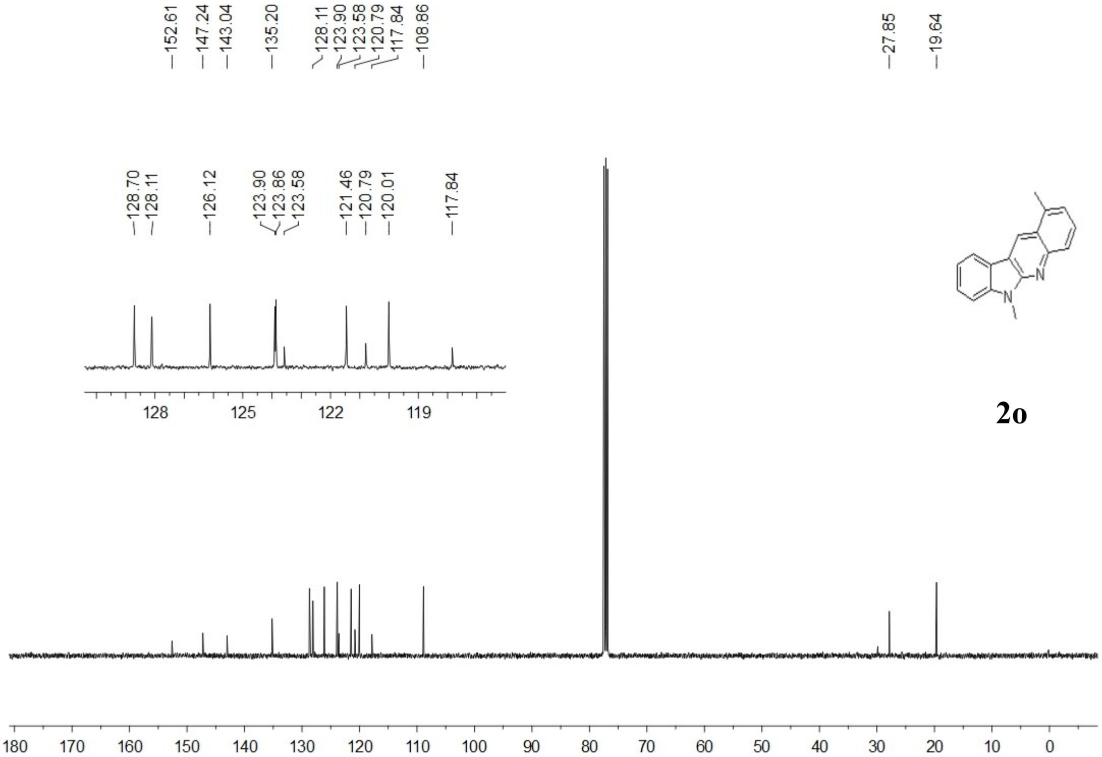
152.61 147.24 143.04 135.20 128.11 123.90 123.58 120.79 117.84 108.86
27.85 19.64
128.70 128.11 126.12 123.90 123.86 123.58 121.46 120.79 120.01 117.84
128 125 122 119
180 170 160 150 140 130 120 110 100 90 80 70 60 50 40 30 20 10 0
N
N

2o

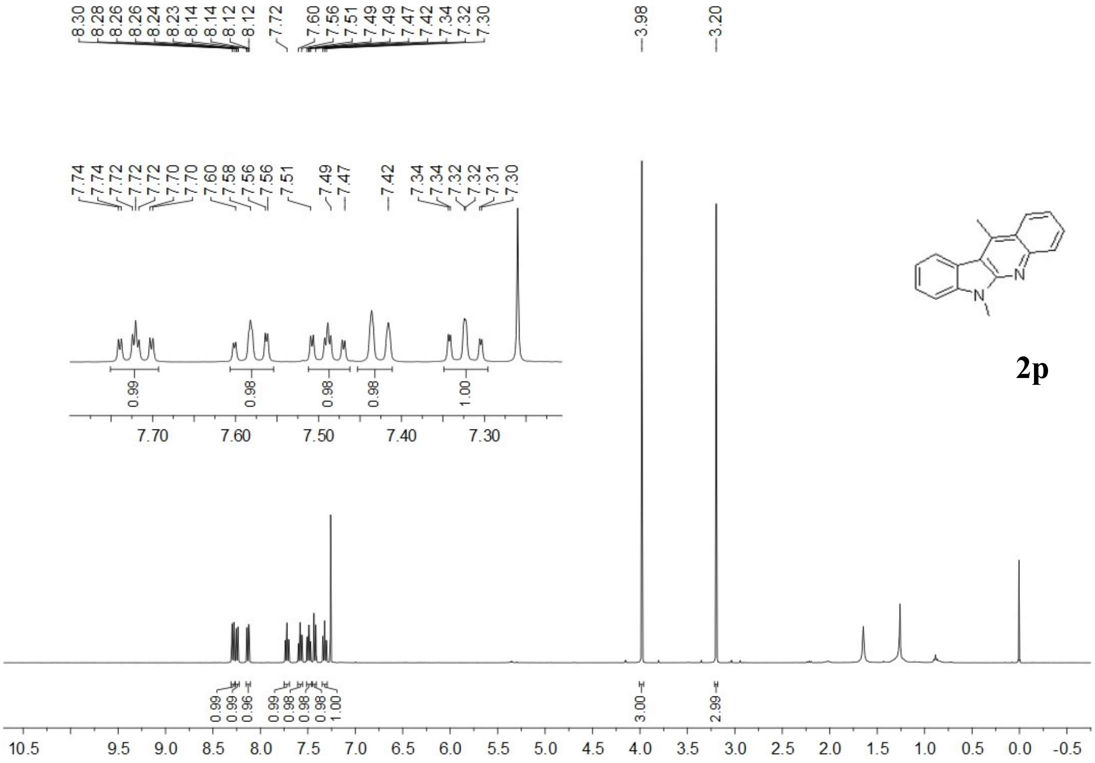
8.30
8.28
8.26
8.26
8.24
8.23
8.14
8.14
8.12
8.12
7.72
7.60
7.56
7.51
7.49
7.49
7.47
7.47
7.42
7.42
7.34
7.32
7.30
3.98
3.20
N
7.70
7.60
7.50
7.40
7.30

2p

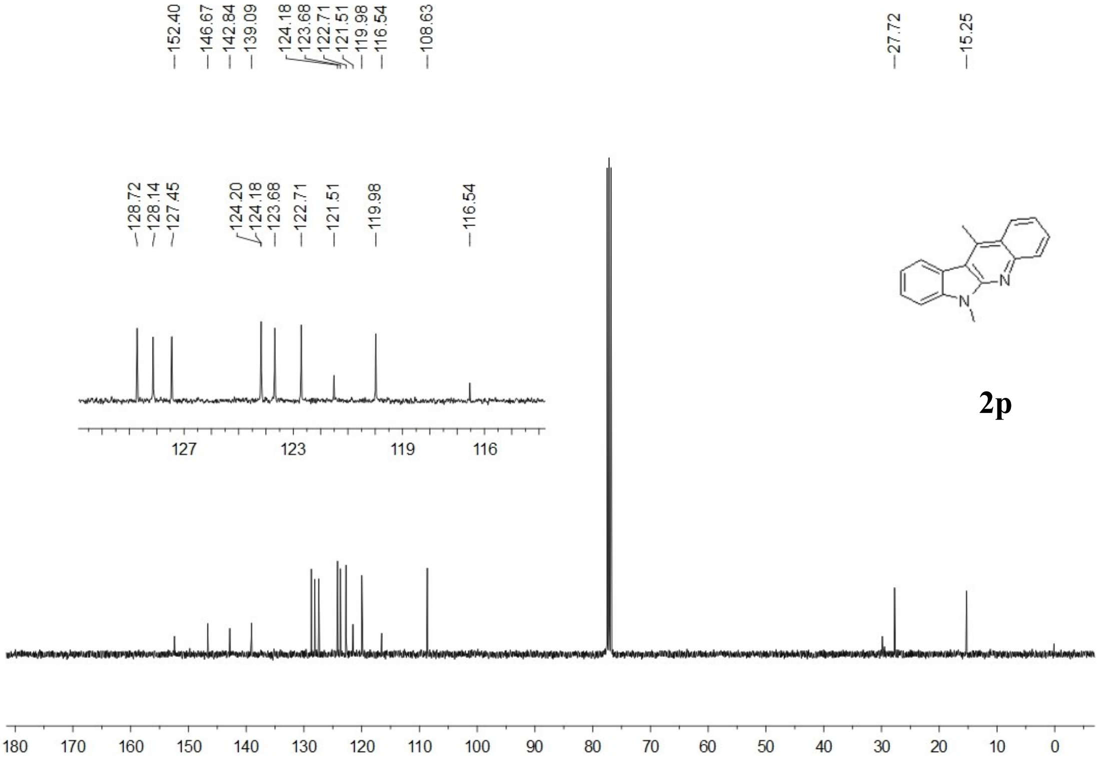
152.40
146.67
142.84
139.09
124.18
123.68
122.71
121.51
119.98
116.54
108.63
27.72
15.25
128.72
128.14
127.45
124.20
124.18
123.68
122.71
121.51
119.98
116.54
N
127
123
119
116

2p

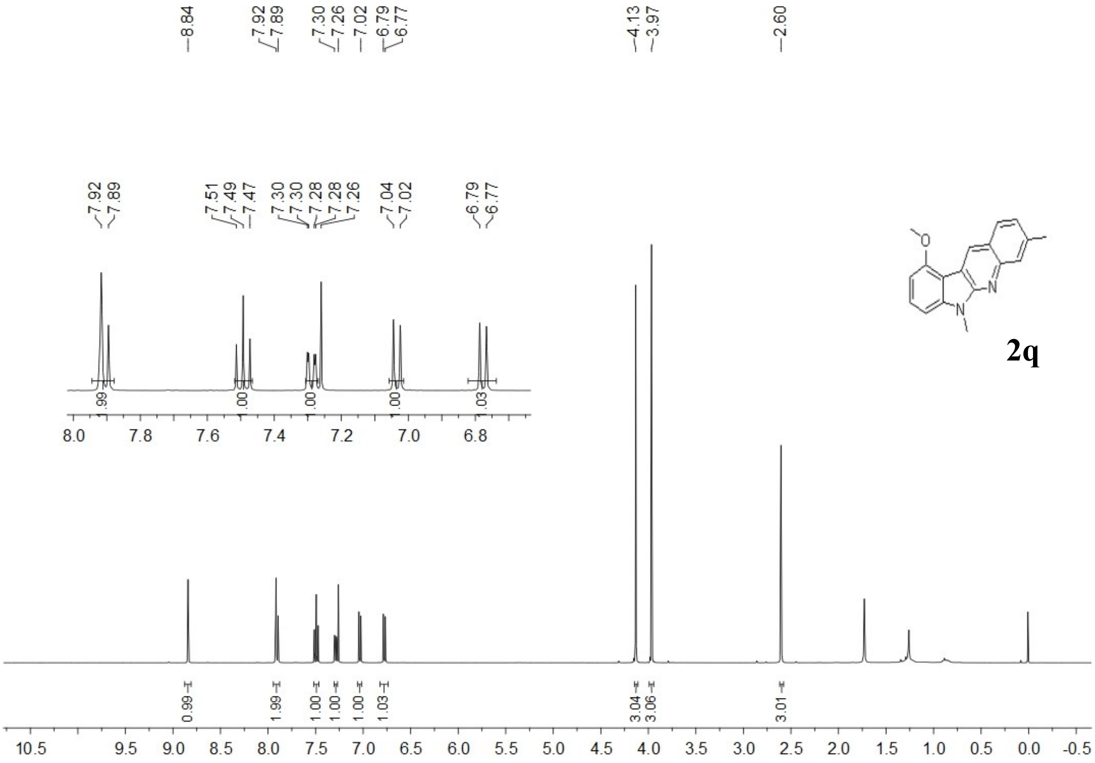
8.84
7.92
7.89
7.30
7.26
7.02
6.79
6.77
4.13
3.97
2.60
7.92
7.89
7.51
7.49
7.47
7.30
7.30
7.28
7.28
7.26
7.04
7.02
6.79
6.77
.99
.00
.00
.00
.03
8.0 7.8 7.6 7.4 7.2 7.0 6.8
2q
0.99
1.99
1.00
1.00
1.00
1.03
3.04
3.06
3.01
10.5 9.5 9.0 8.5 8.0 7.5 7.0 6.5 6.0 5.5 5.0 4.5 4.0 3.5 3.0 2.5 2.0 1.5 1.0 0.5 0.0 -0.5

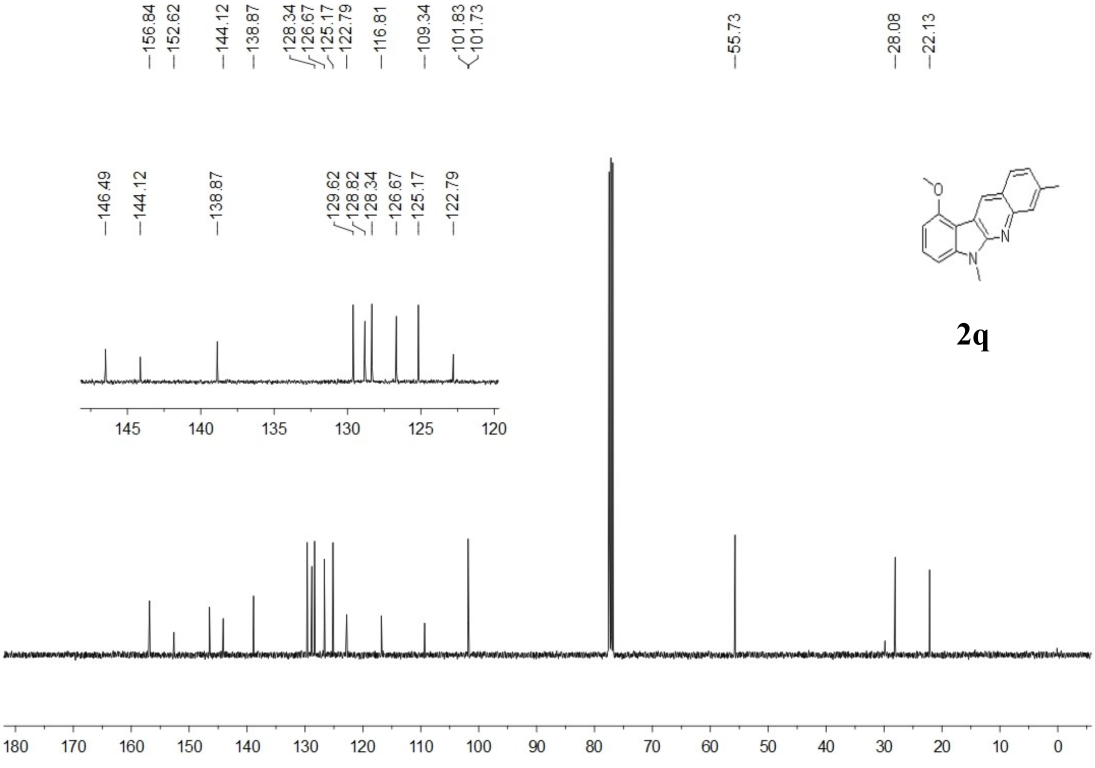
156.84
152.62
144.12
138.87
128.34
126.67
125.17
122.79
116.81
109.34
101.83
101.73
55.73
28.08
22.13
146.49
144.12
138.87
129.62
128.82
128.34
126.67
125.17
122.79
145 140 135 130 125 120
2q
180 170 160 150 140 130 120 110 100 90 80 70 60 50 40 30 20 10 0

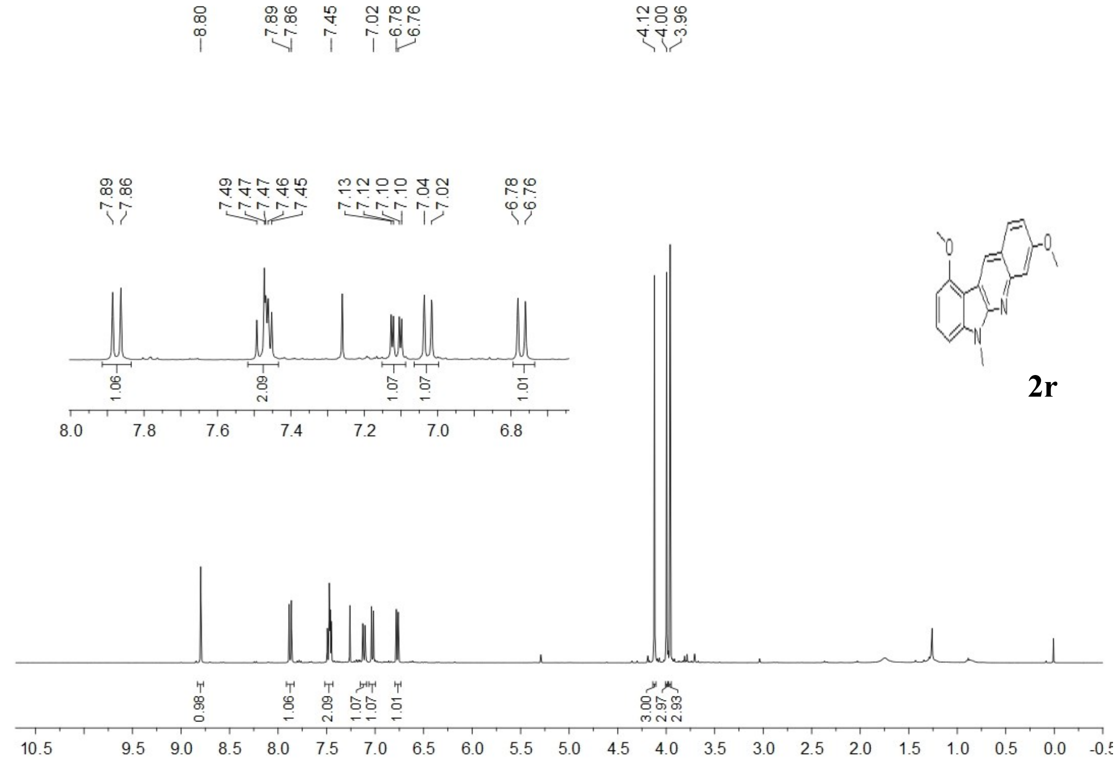
8.80
7.89
7.86
7.45
7.02
6.78
6.76
4.12
4.00
3.96
7.89
7.86
7.49
7.47
7.47
7.46
7.45
7.13
7.12
7.10
7.10
7.04
7.02
6.78
6.76
1.06
2.09
1.07
1.07
1.01
0.98
1.06
2.09
1.07
1.07
1.01
3.00
2.97
2.93

2r

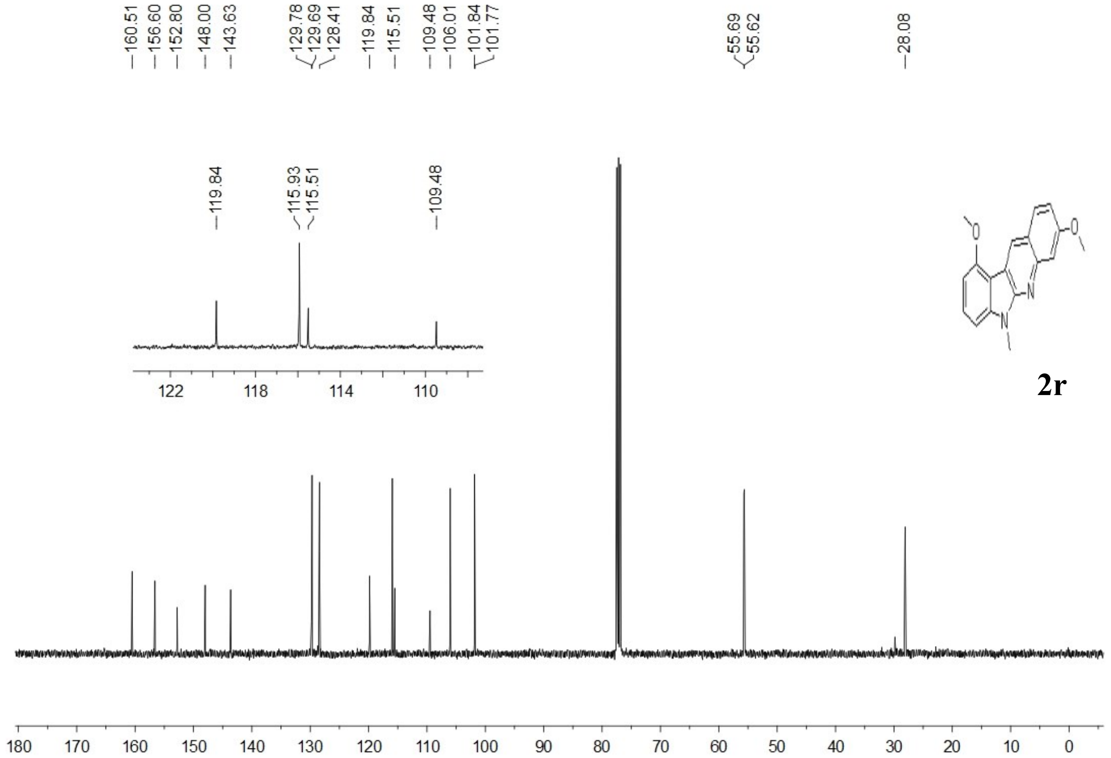
160.51
156.60
152.80
148.00
143.63
129.78
129.69
128.41
119.84
115.51
109.48
106.01
101.84
101.77
55.69
55.62
28.08
119.84
115.93
115.51
109.48

2r

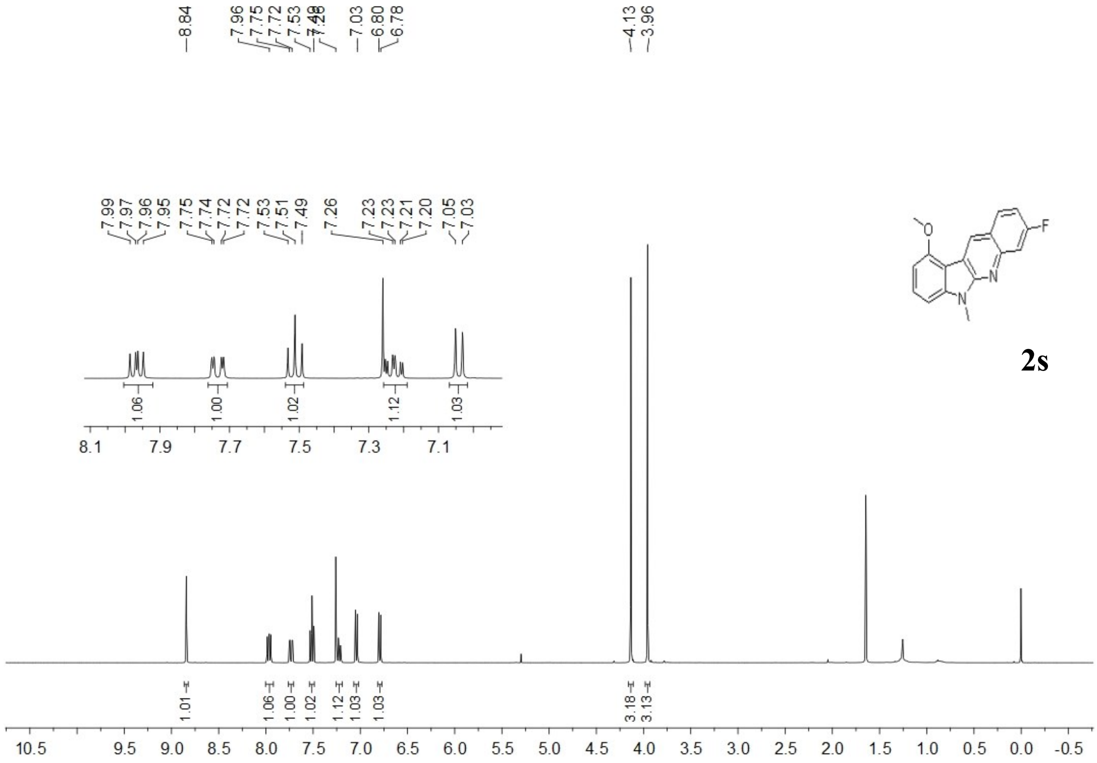

2s

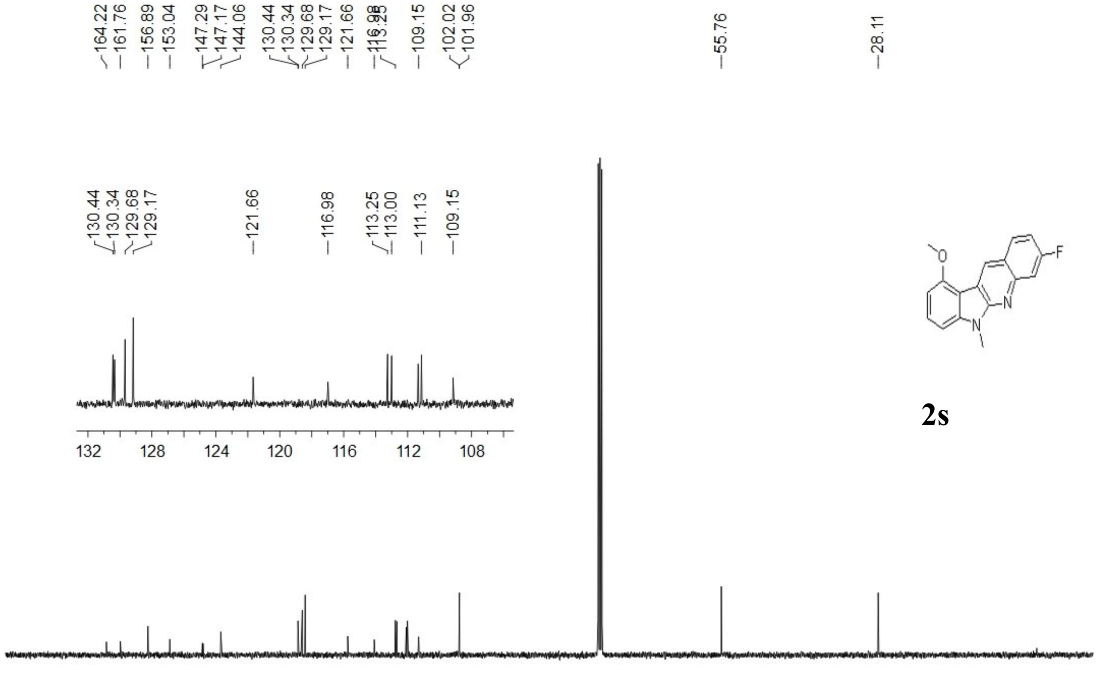

2s

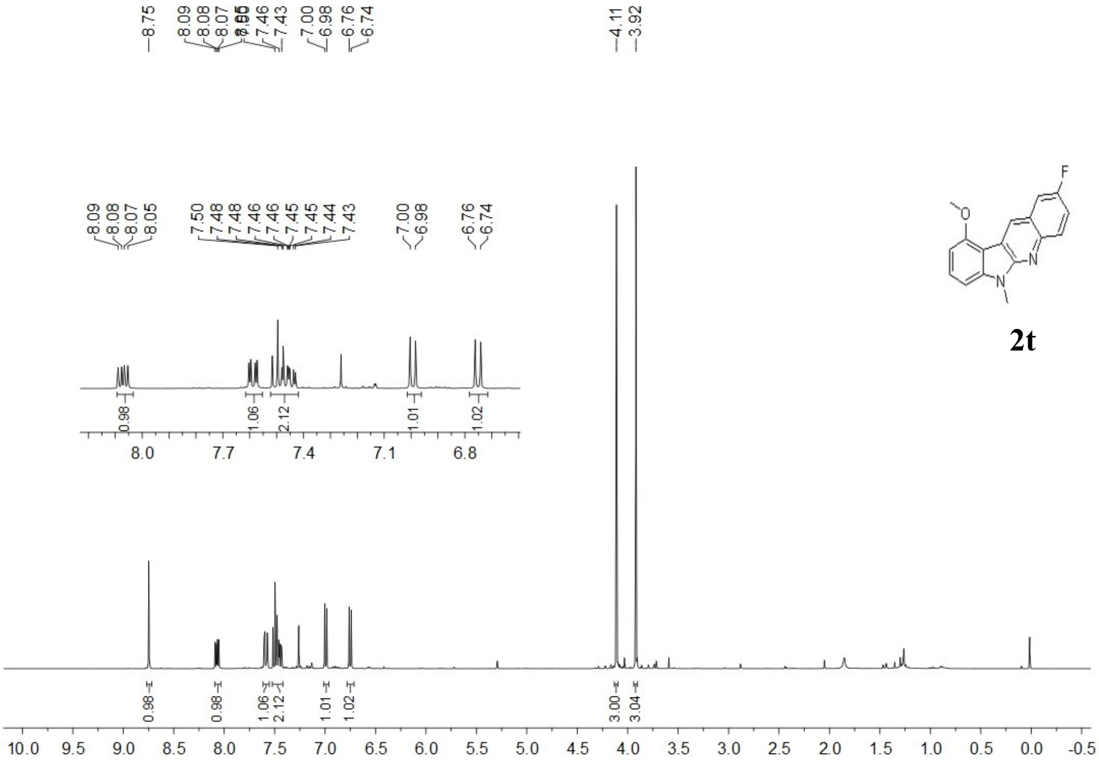
8.75
8.09
8.08
8.07
7.46
7.43
7.00
6.98
6.76
6.74
4.11
3.92
8.09
8.08
8.07
8.05
7.50
7.48
7.48
7.46
7.46
7.45
7.45
7.44
7.43
7.00
6.98
6.76
6.74
0.98
1.06
2.12
1.01
1.02
8.0
7.7
7.4
7.1
6.8
0.98
0.98
1.06
2.12
1.01
1.02
3.00
3.04
10.0 9.5 9.0 8.5 8.0 7.5 7.0 6.5 6.0 5.5 5.0 4.5 4.0 3.5 3.0 2.5 2.0 1.5 1.0 0.5 0.0 -0.5

2t

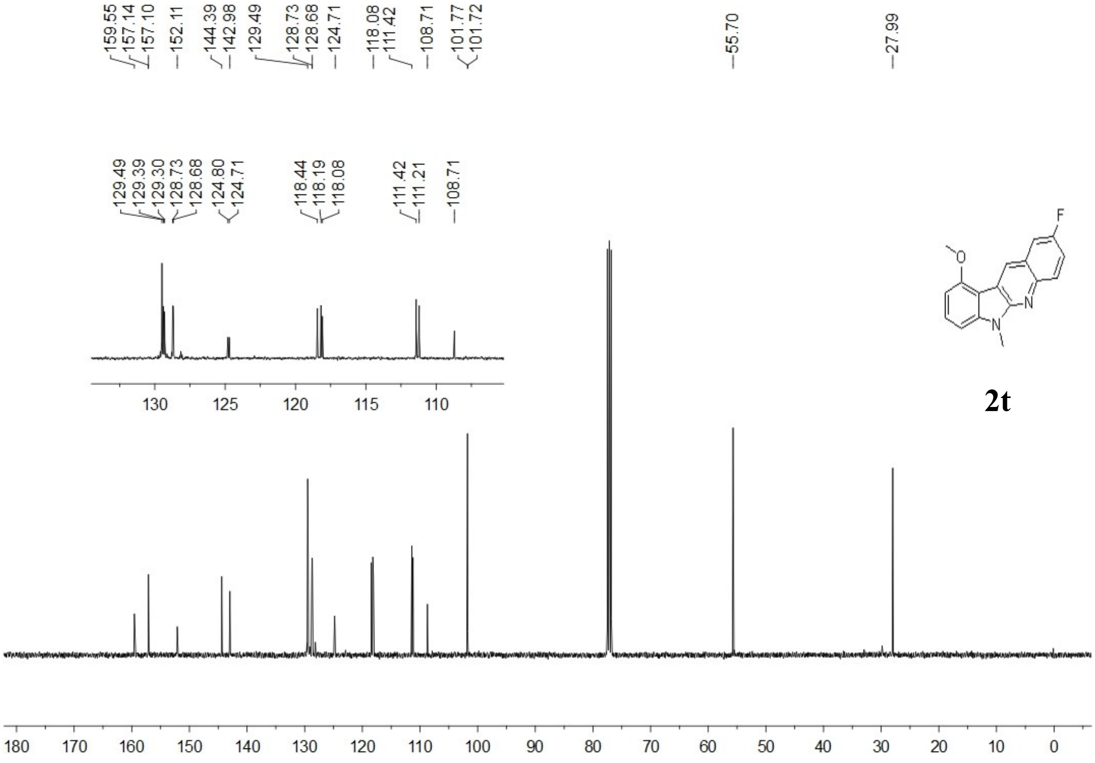
159.55
157.14
157.10
152.11
144.39
142.98
129.49
128.73
128.68
124.71
118.08
111.42
108.71
101.77
101.72
55.70
27.99
129.49
129.39
129.30
128.73
128.68
124.80
124.71
118.44
118.19
118.08
111.42
111.21
108.71
130 125 120 115 110
180 170 160 150 140 130 120 110 100 90 80 70 60 50 40 30 20 10 0

2t

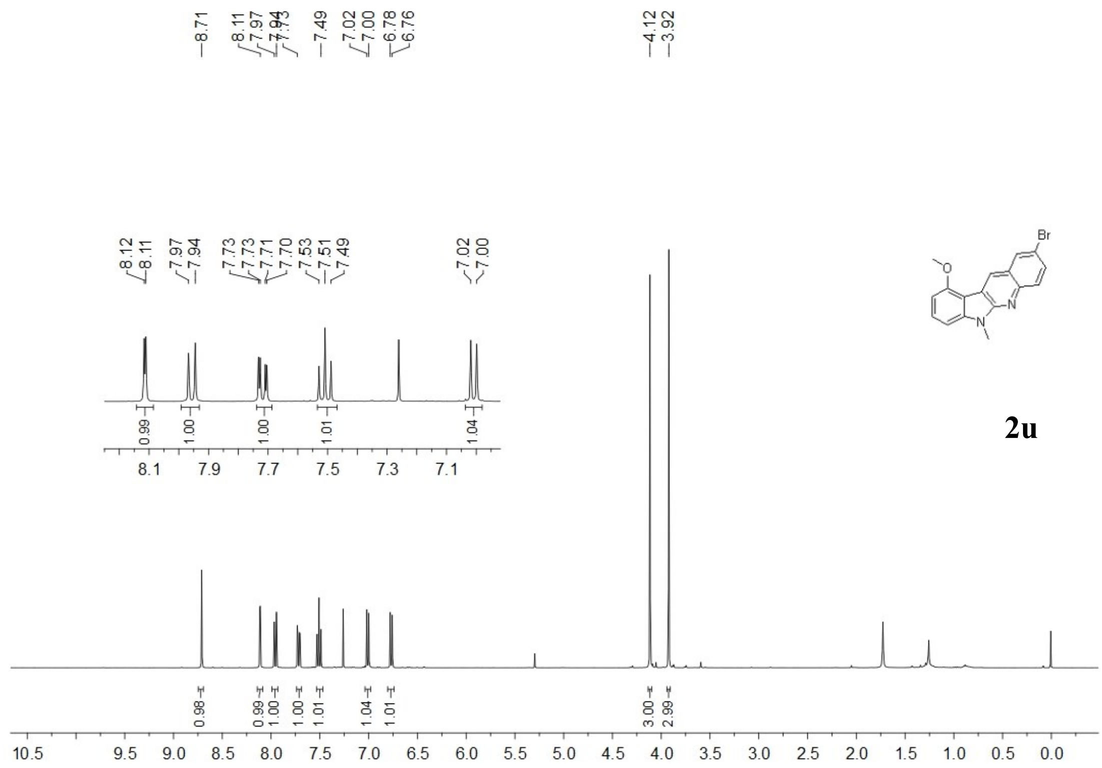
8.71
8.11
7.97
7.94
7.73
7.49
7.02
7.00
6.78
6.76
4.12
3.92
8.12
8.11
7.97
7.94
7.73
7.73
7.71
7.70
7.53
7.51
7.49
7.02
7.00
Br
N
N
0.99
1.00
1.00
1.01
1.04
8.1
7.9
7.7
7.5
7.3
7.1
0.98
0.99
1.00
1.00
1.01
1.04
1.01
3.00
2.99
10.5
9.5
9.0
8.5
8.0
7.5
7.0
6.5
6.0
5.5
5.0
4.5
4.0
3.5
3.0
2.5
2.0
1.5
1.0
0.5
0.0

2u

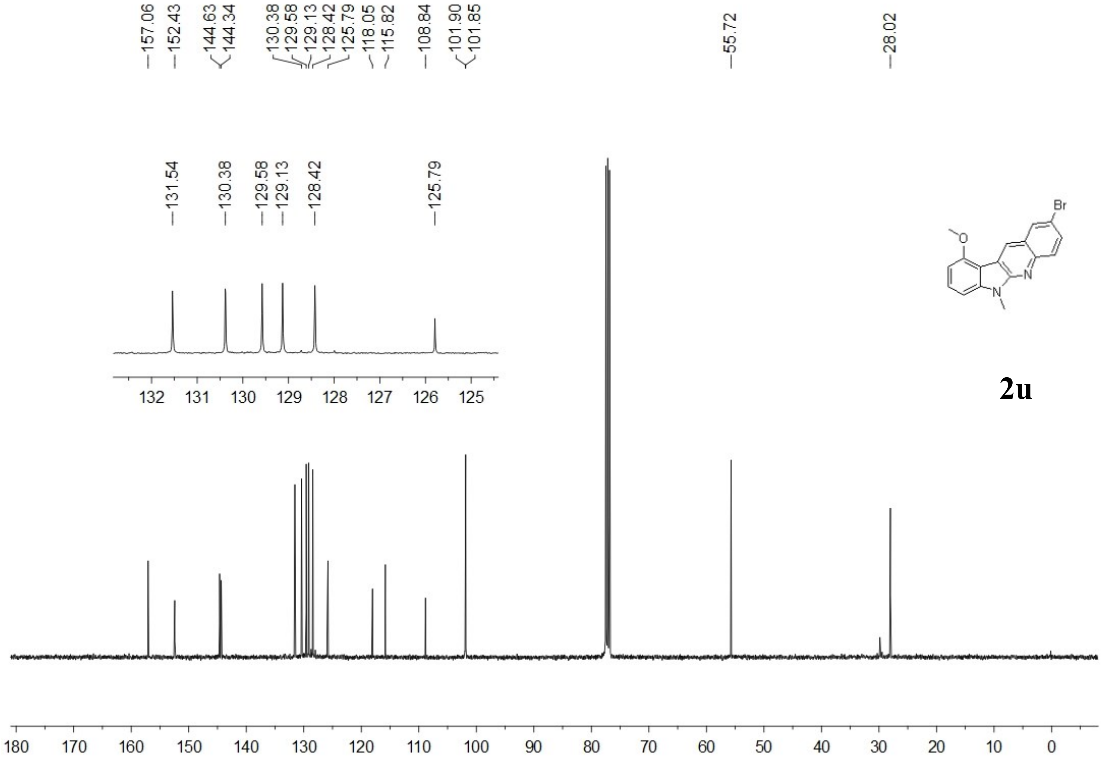
157.06
152.43
144.63
144.34
130.38
129.58
129.13
128.42
125.79
118.05
115.82
108.84
101.90
101.85
55.72
28.02
131.54
130.38
129.58
129.13
128.42
125.79
132
131
130
129
128
127
126
125
Br
N
N
180
170
160
150
140
130
120
110
100
90
80
70
60
50
40
30
20
10
0

2u

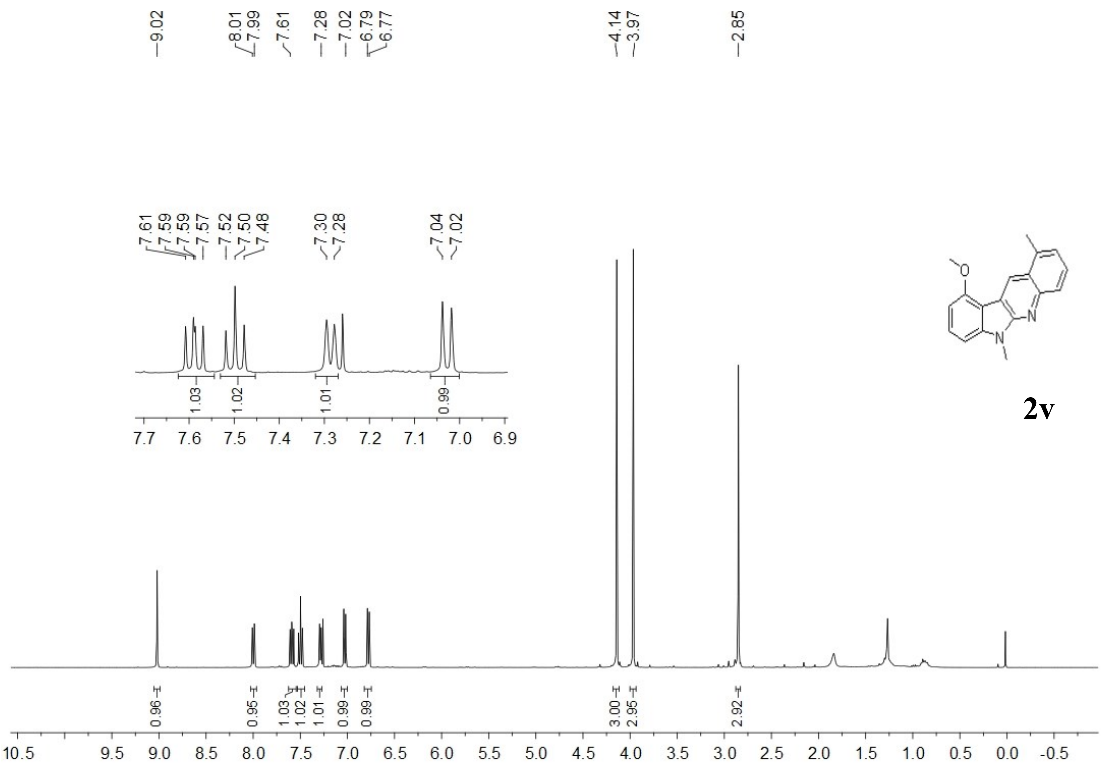
9.02
8.01
7.99
7.61
7.28
7.02
6.79
6.77
4.14
3.97
2.85
7.61
7.59
7.59
7.57
7.52
7.50
7.48
7.30
7.28
7.04
7.02
1.03
1.02
1.01
0.99
7.7 7.6 7.5 7.4 7.3 7.2 7.1 7.0 6.9
0.96
0.95
1.03
1.02
1.01
0.99
0.99
3.00
2.95
2.92
10.5 9.5 9.0 8.5 8.0 7.5 7.0 6.5 6.0 5.5 5.0 4.5 4.0 3.5 3.0 2.5 2.0 1.5 1.0 0.5 0.0 -0.5

2v

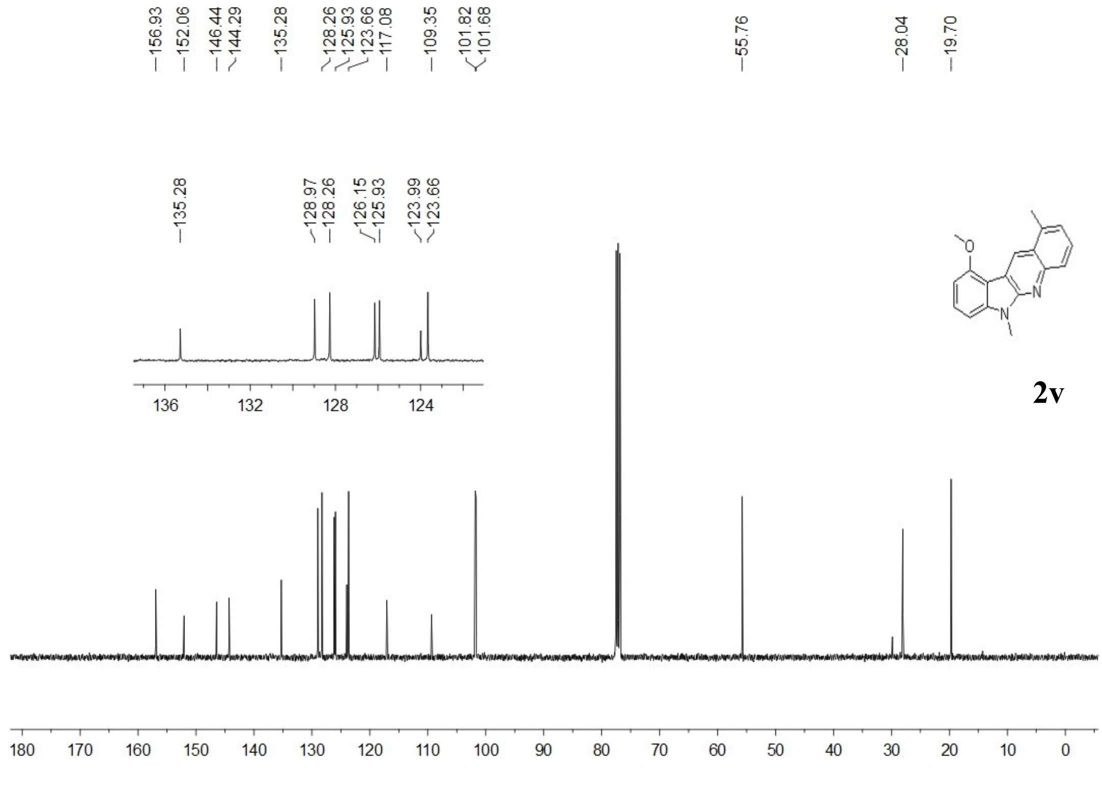
156.93
152.06
146.44
144.29
135.28
128.26
125.93
123.66
117.08
109.35
101.82
101.68
55.76
28.04
19.70
135.28
128.97
128.26
126.15
125.93
123.99
123.66
136 132 128 124
180 170 160 150 140 130 120 110 100 90 80 70 60 50 40 30 20 10 0

2v

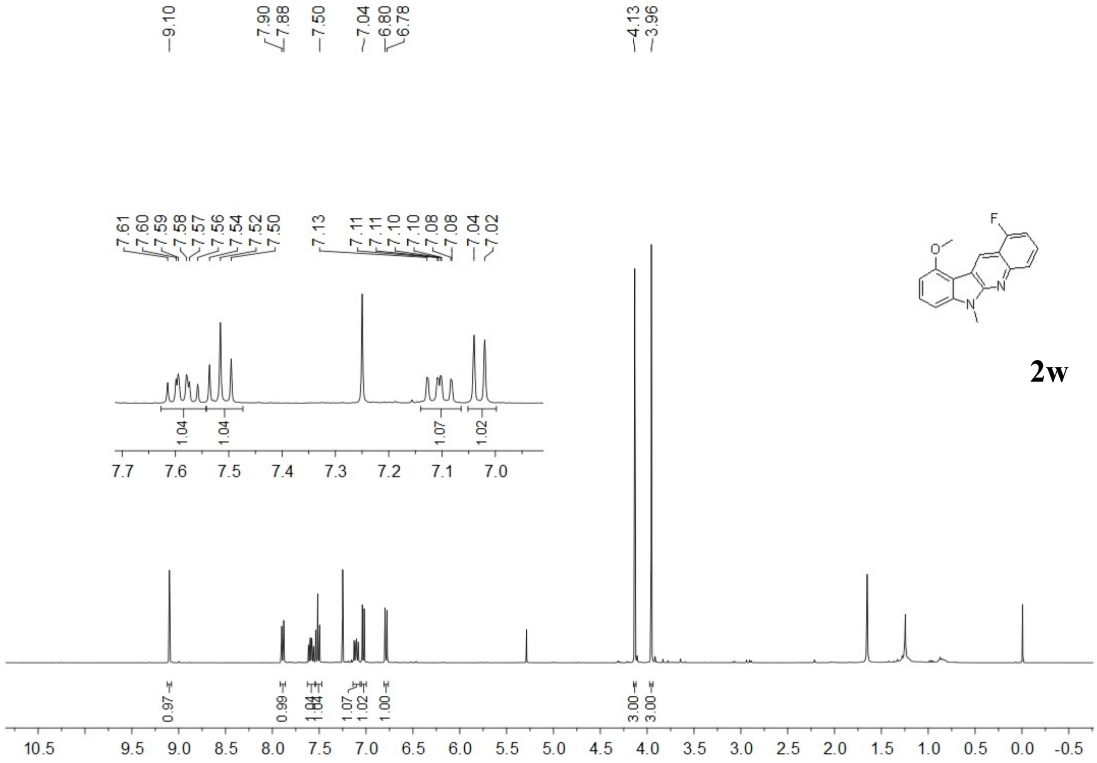
9.10
7.90
7.88
7.50
7.04
6.80
6.78
4.13
3.96
7.61
7.60
7.59
7.58
7.57
7.56
7.54
7.52
7.50
7.13
7.11
7.11
7.10
7.10
7.08
7.08
7.04
7.02
F
N
N
2w
0.97
0.99
1.04
1.04
1.07
1.02
1.00
3.00
3.00
10.5 9.5 9.0 8.5 8.0 7.5 7.0 6.5 6.0 5.5 5.0 4.5 4.0 3.5 3.0 2.5 2.0 1.5 1.0 0.5 0.0 -0.5
7.7 7.6 7.5 7.4 7.3 7.2 7.1 7.0

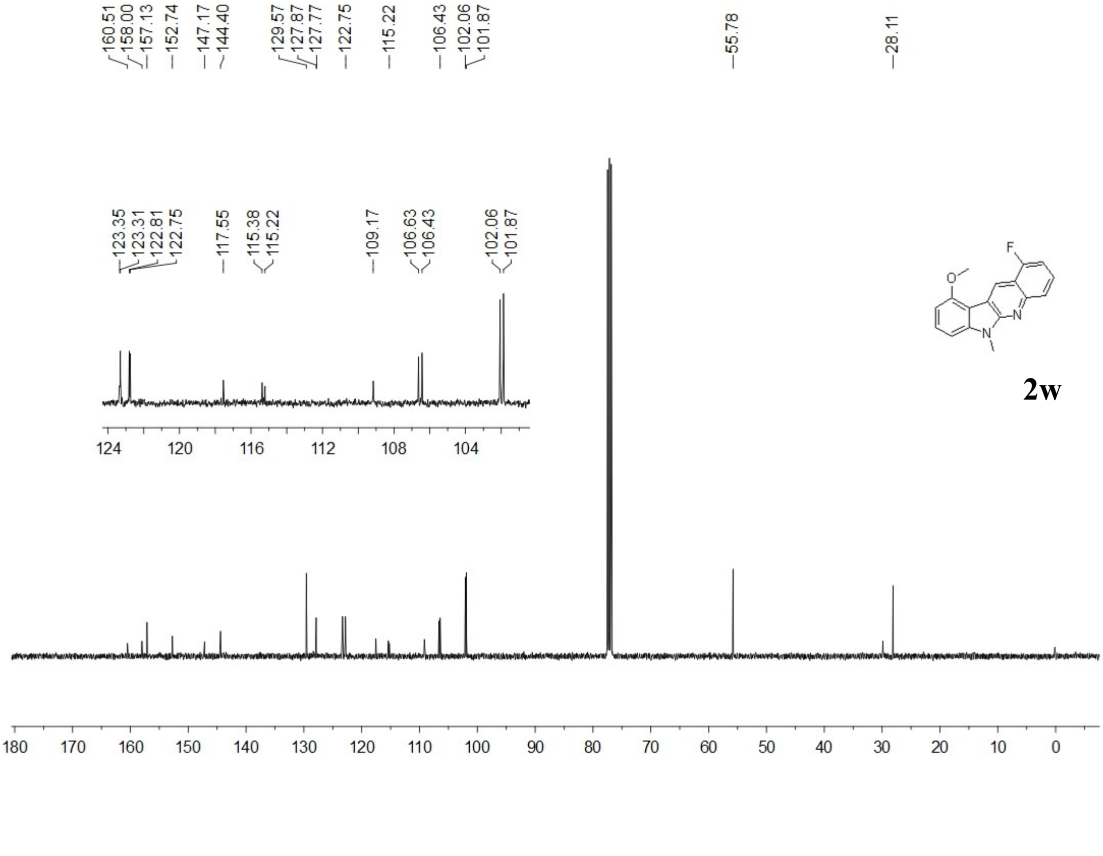
160.51
158.00
157.13
152.74
147.17
144.40
129.57
127.87
127.77
122.75
115.22
106.43
102.06
101.87
55.78
28.11
123.35
123.31
122.81
122.75
117.55
115.38
115.22
109.17
106.63
106.43
102.06
101.87
F
N
N
2w
124 120 116 112 108 104
180 170 160 150 140 130 120 110 100 90 80 70 60 50 40 30 20 10 0

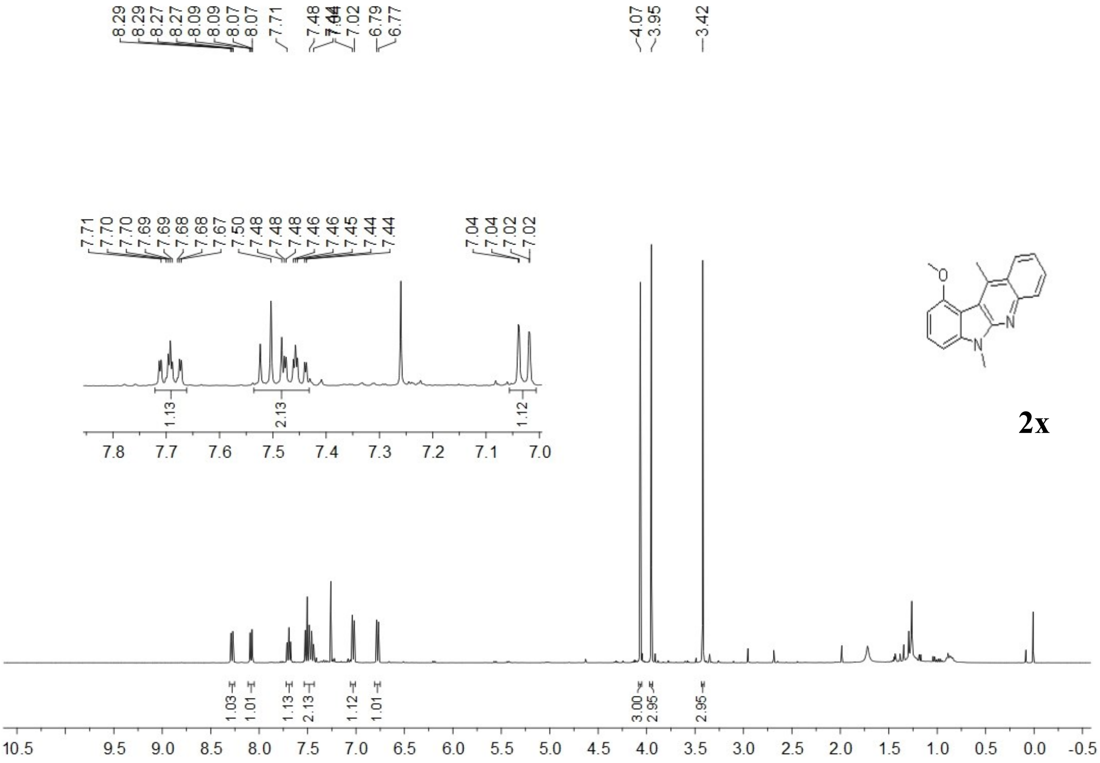
8.29 8.29 8.27 8.27 8.09 8.09 8.07 8.07 7.71 7.48 7.44 7.44 7.02 6.79 6.77
4.07 3.95 3.42
7.71 7.70 7.70 7.69 7.69 7.68 7.68 7.67 7.50 7.48 7.48 7.48 7.46 7.46 7.45 7.44 7.44
7.04 7.04 7.02 7.02
1.13 2.13 1.12
7.8 7.7 7.6 7.5 7.4 7.3 7.2 7.1 7.0
1.03 1.01 1.13 2.13 1.12 1.01 3.00 2.95 2.95
10.5 9.5 9.0 8.5 8.0 7.5 7.0 6.5 6.0 5.5 5.0 4.5 4.0 3.5 3.0 2.5 2.0 1.5 1.0 0.5 0.0 -0.5

2x

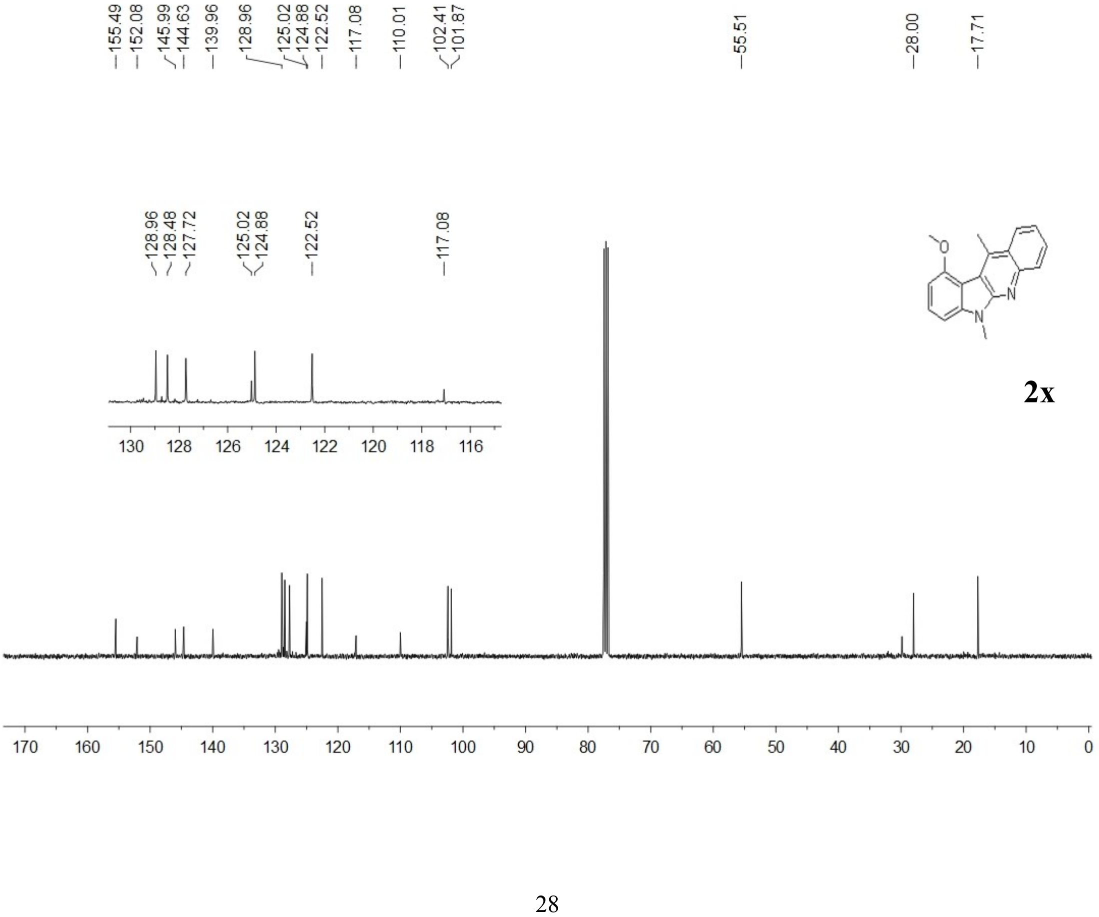
155.49 152.08 145.99 144.63 139.96 128.96 125.02 124.88 122.52 117.08 110.01 102.41 101.87
55.51 28.00 17.71
128.96 128.48 127.72 125.02 124.88 122.52 117.08
130 128 126 124 122 120 118 116
170 160 150 140 130 120 110 100 90 80 70 60 50 40 30 20 10 0

2x
